# Supplementary material for: pKID Binds to KIX via an Unstructured Transition State with Nonnative Interactions
Source: Biophys J. 2017 Dec 19;113(12):2713–22. doi: 10.1016/j.bpj.2017.10.016 (PMC5770965; doi:10.1016/j.bpj.2017.10.016)
Supplement: Document S1. Figs. S1–S7 and Tables S1 and S2 [file mmc1.pdf]

Biophysical Journal, Volume *113*

Supplemental Information

pKID Binds to KIX via an Unstructured Transition State with Nonnative Interactions

Liza Dahal, Tristan O.C. Kwan, Sarah L. Shammas, and Jane Clarke

Supplementary Tables

Table S1. Kinetic and thermodynamic rate constants for the binding of wild-type and interface mutants of pKID to KIX. The errors for $k_{ass,fast}$, k_{diss} and K_d represent errors from the fit. Φ-values were calculated using both kinetic and equilibrium measurements and the errors propagated using standard equations.

pKID	$k_{ass,fast}$ (μM^{-1}s^{-1})	k_{diss} (s^{-1})	K_{dEqub} (μM)	K_{dKin} (μM)	$\Delta\Delta G_{Equb}$ (kcal.mol^{-1})	$\Delta\Delta G_{Kin}$ (kcal.mol^{-1})	Φ_{Equb}	Φ_{Kin}
**Interface mutants**								
Wild-type	7.3±0.3	0.81±0.01	0.11±0.02	0.11±0.02	-	-	-	-
S121A	6.9±1.2	0.71±0.01	0.12±0.02	0.10±0.02	0.05±0.14	-0.04±0.10	0.65±2.75*	-0.75±4.14*
R124A	8.4±1.6	0.51±0.1	0.06±0.01	0.06±0.02	-0.34±0.14	-0.34±0.16	0.23±0.34	0.23±0.26
R125A	6.3±0.5	2.17±0.04	0.22±0.02	0.34±0.03	0.39±0.11	0.64±0.05	0.21±0.14	0.13±0.07
L128A	7.9±0.3	15.7±1.3	2.49±0.06	2.00±0.16	1.75±0.10	1.62±0.05	-0.03±0.02	-0.03±0.02
R131A	9.5±1	7.66±1.04	1.23±0.12	0.81±0.14	1.36±0.12	1.12±0.10	-0.11±0.05	-0.13±0.07
ΔpS133	7.6±0.8	56.7±7.5	28.7±1.2	7.5±1.3	3.13±0.10	2.37±0.10	-0.01±0.02	-0.01±0.03
Y134A	†	†	110.6±9.8	†	3.89±0.11	†	†	†
I137A	4.5±0.5	43.9±7.2	21.6±0.50	9.73±1.92	2.97±0.10	2.52±0.11	0.09±0.02	0.11±0.02
L138A	2.8±1.1	13.2±1.4	6.30±0.30	4.73±1.92	2.28±0.11	2.11±0.23	0.24±0.10	0.26±0.08
D140A	5.3±0.7	2.06±0.09	0.26±0.04	0.39±0.05	0.48±0.13	0.70±0.08	0.37±0.19	0.26±0.08
S142A	11.3±1	0.83±0.03	0.12±0.01	0.07±0.01	0.05±0.11	-0.23±0.06	-5.20±11.6*	1.06±0.10*

*Mutants where Φ-values are unreliable and not reported in the main text as they show $\Delta\Delta G_{Equb}$ and/or $\Delta\Delta G_{Kin} < 0.34$ kcal mol^{-1}. (See main paper for discussion.)
† Kinetic measurements could not be obtained, as good signal change upon binding was not observed.

Table S2. Kinetic and thermodynamic rate constants for the binding of solvent exposed mutants of pKID to KIX. The errors for $k_{ass,fast}$, k_{diss} and K_d represent errors from the fit. Φ-values were calculated using both kinetic and equilibrium measurements and the errors propagated using standard equations. ΔΔG values for Ala-Gly composite mutations are shown.

**pKID**	$k_{ass,fast}$ **(μM^{-1}s^{-1})**	k_{diss} **(s^{-1})**	K_{dEqub} **(μM)**	K_{dKin} **(μM)**	**ΔΔG_{Equb} (kcal.mol^{-1})**	**ΔΔG_{Kin} (kcal.mol^{-1})**	**Φ$_{Equb}$**	**Φ$_{Kin}$**
**Solvent Exposed mutants**								
T119A	7.3±0.7	0.88±0.01	0.15±0.02	0.12±0.01	0.17±0.13	0.05±0.06	-	-
A119G	7.1±1.1	0.67±0.02	0.08±0.02	0.09±0.01	-0.35±0.16	-0.14±0.10	-0.04±0.29*	0.11±0.83*
E126A	10.5±1.7	0.97±0.01	0.17±0.01	0.09±0.01	0.24±0.11	-0.10±0.09	-	-
A126G	7.6±0.9	2.09±0.02	0.25±0.02	0.28±0.03	0.22±0.06	0.61±0.11	0.84±0.56*	0.30±0.13*
P132A	7.7±0.4	0.99±0.05	0.07±0.02	0.13±0.01	-0.25±0.19	0.08±0.05	-	-
A132G	8.3±0.2	3.22±0.03	0.44±0.03	0.39±0.01	1.03±0.17	0.62±0.04	-0.04±0.03	-0.07±0.06
R135A	10.5±0.6	2.50±0.20	0.10±0.02	0.24±0.02	-0.05±0.15	0.43±0.06	-	-
A135G	8±0.4	10.1±0.30	1.16±0.05	1.26±0.07	1.38±0.12	0.94±0.06	0.11±0.03	0.16±0.04
K136A	10.2±0.9	0.94±0.03	0.10±0.02	0.09±0.01	-0.05±0.15	-0.10±0.06	-	-
A136G	8.3±1.5	10.5±0.80	2.16±0.11	1.27±0.25	1.73±0.12	1.47±0.12	0.07±0.07	0.08±0.07
S143A	9.7±1	0.71±0.01	0.07±0.01	0.07±0.01	-0.25±0.13	-0.23±0.06	-	-
A143G	6.3±0.6	1.20±0.10	0.09±0.01	0.19±0.02	0.14±0.10	0.54±0.09	1.72±1.36*	0.45±0.09*

*Mutants where Φ-values are unreliable and not reported in the main text as they show ΔΔG_{Equb} and/or ΔΔG_{Kin} < 0.34 kcal mol^{-1}. (See main paper for discussion.)

Supplementary Figures

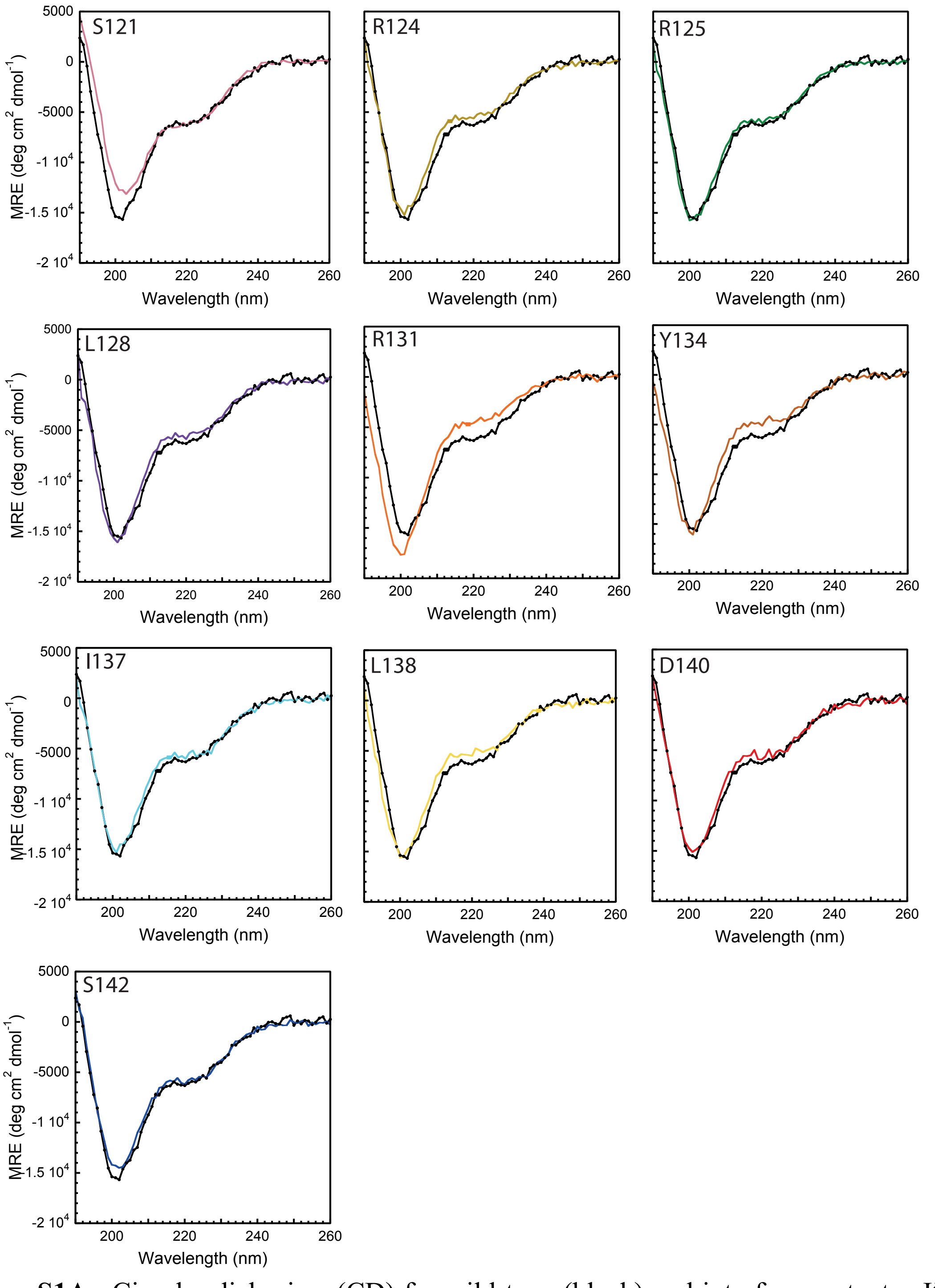

Figure S1A. Circular dichorism (CD) for wild-type (black) and interface mutants pKID in absence of KIX.

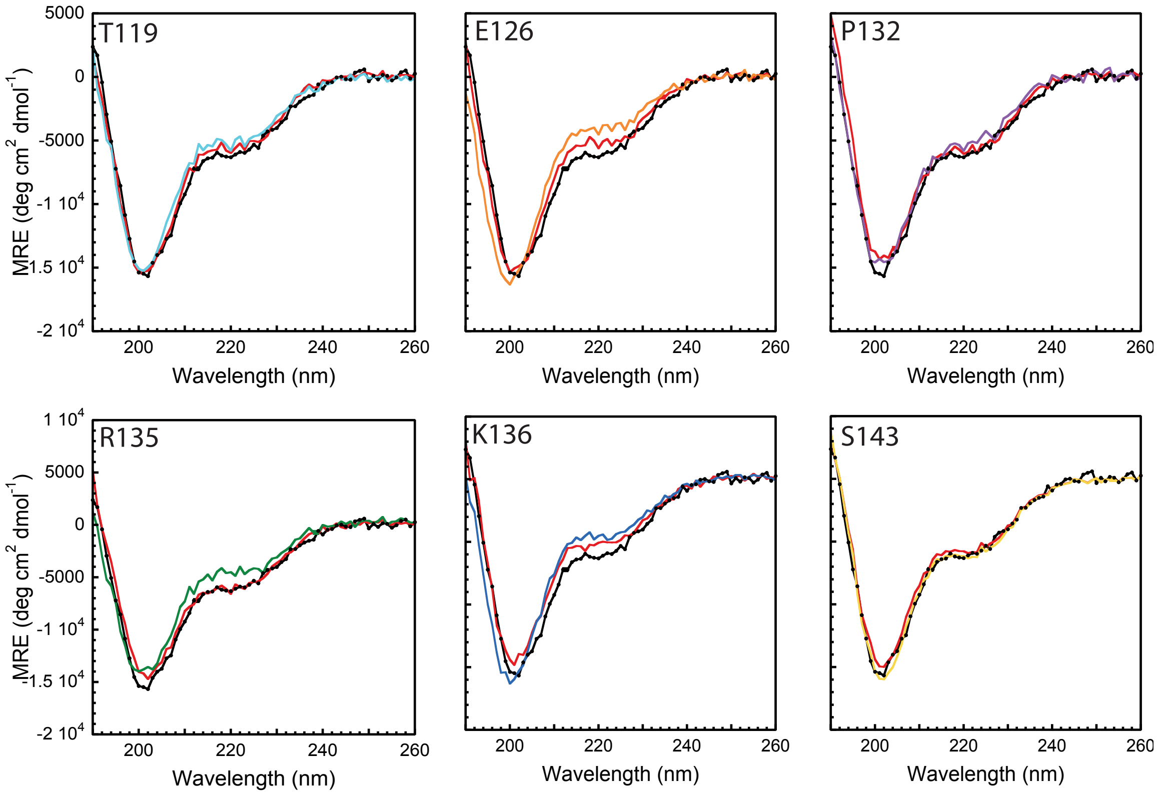

Figure S1B. Circular dichorism (CD) for wild-type (black) and solvent exposed mutants (Ala and Gly) of pKID in absence of KIX. Ala mutations are shown in red and Gly mutants are shown as labeled.

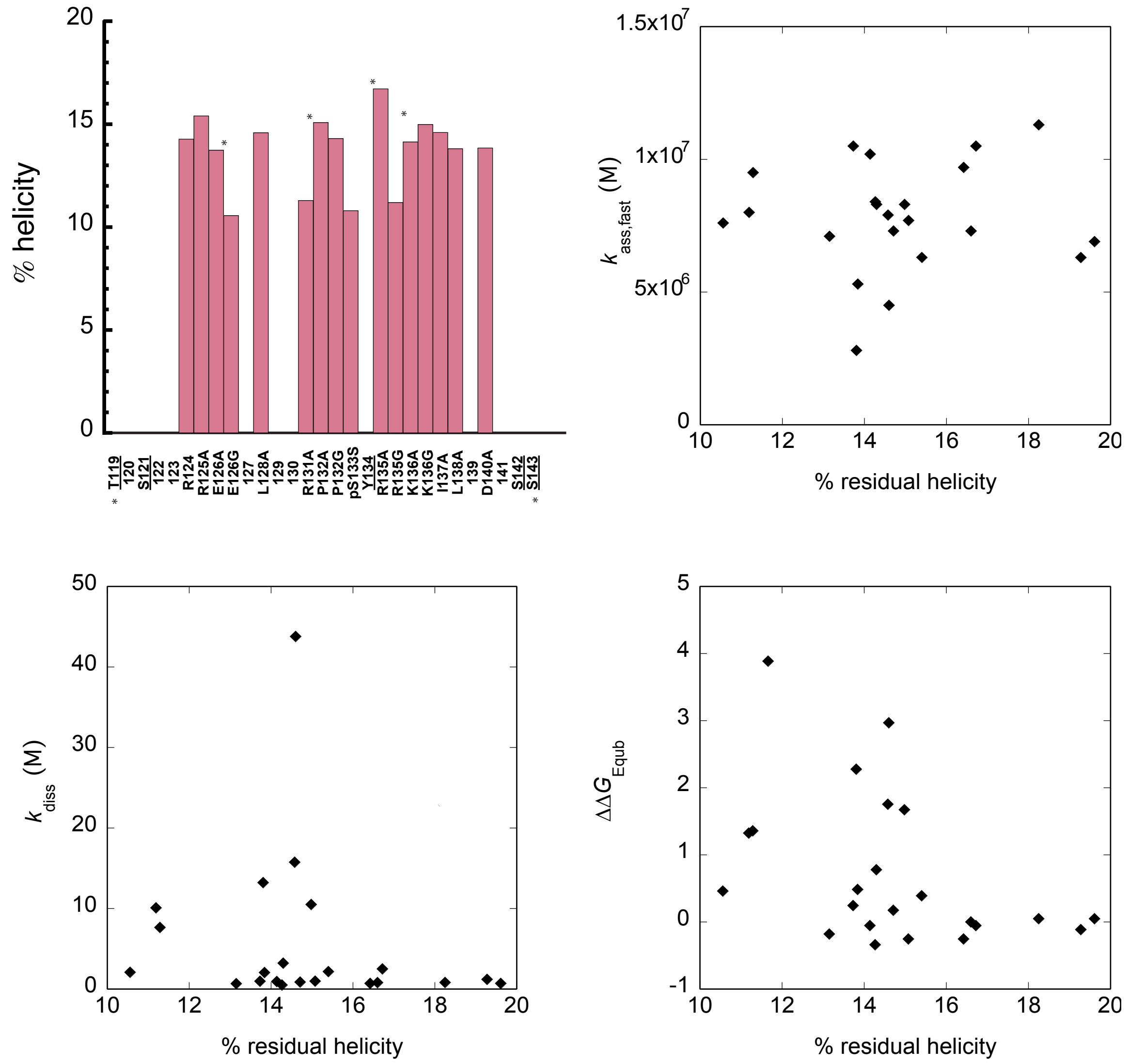

Figure S2. Percentage (%) residual helicity of pKID mutants. (Top left panel) % helicity of interface and solvent exposed pKID mutants used to calculate the Φ values are shown as bar charts. The * on top of the bar highlights the solvent exposed (Ala-Gly) mutants in the bar chart. There is no detectable relationship between residual helicity and any biophysical parameters: (Top right panel) fast association kinetic rate ($k_{ass,fast}$) plotted against % helicity for all pKID mutants. (Bottom left panel) dissociation kinetic rate (k_{diss}) plotted against % helicity for all pKID mutants. (Bottom right panel) $\Delta\Delta G_{Equb}$ plotted against % helicity for all pKID mutants.

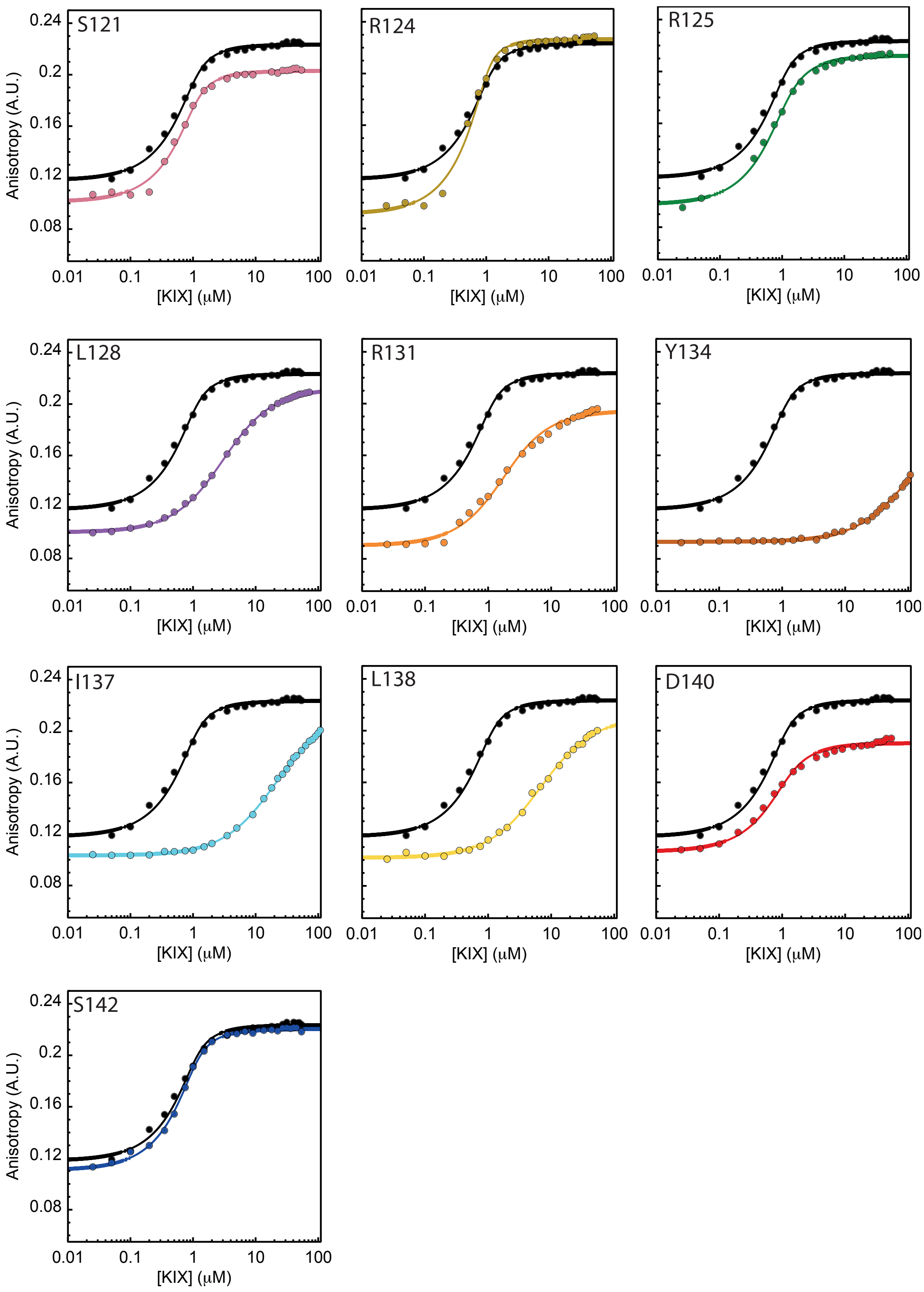

Figure S3A. Equilibrium anisotropy binding curves for the interface mutants of pKID. Wild-type is shown in black.

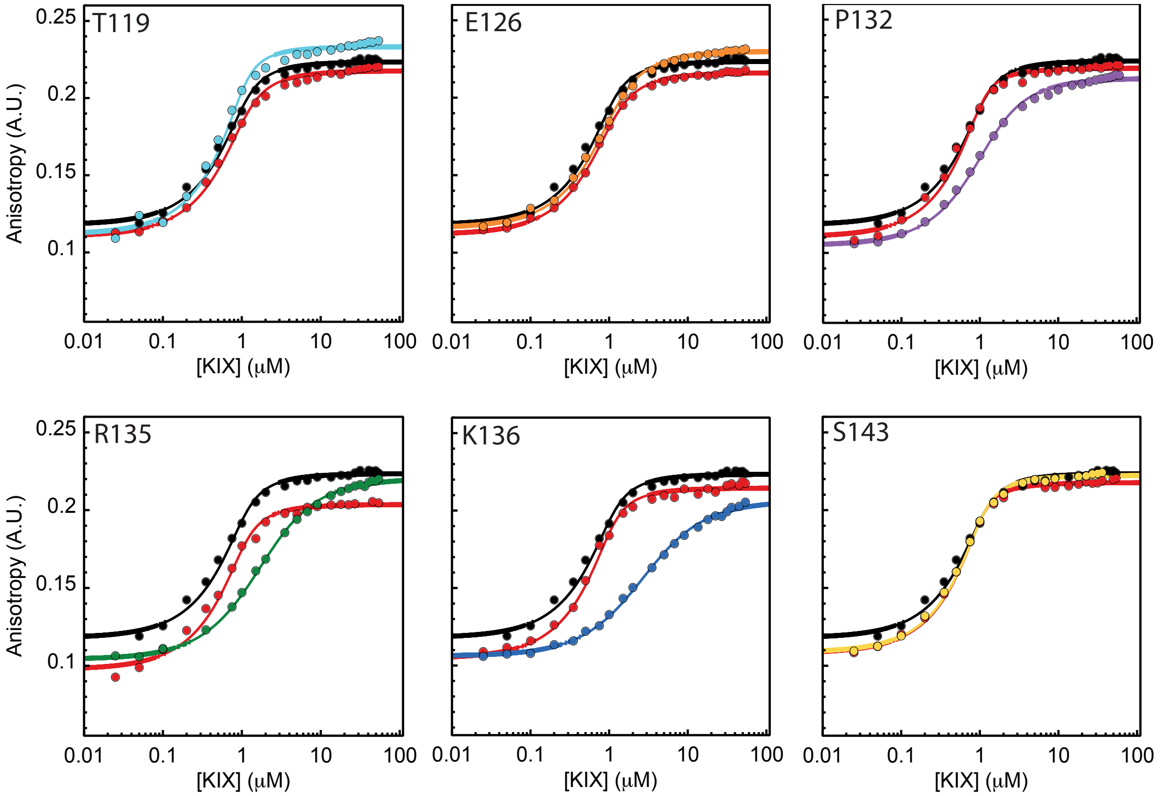

Figure S3B. Equilibrium anisotropy binding curves for the solvent exposed mutants of pKID. Wild-type is shown in black and all Ala mutants are shown in red. Gly mutants are as labeled.

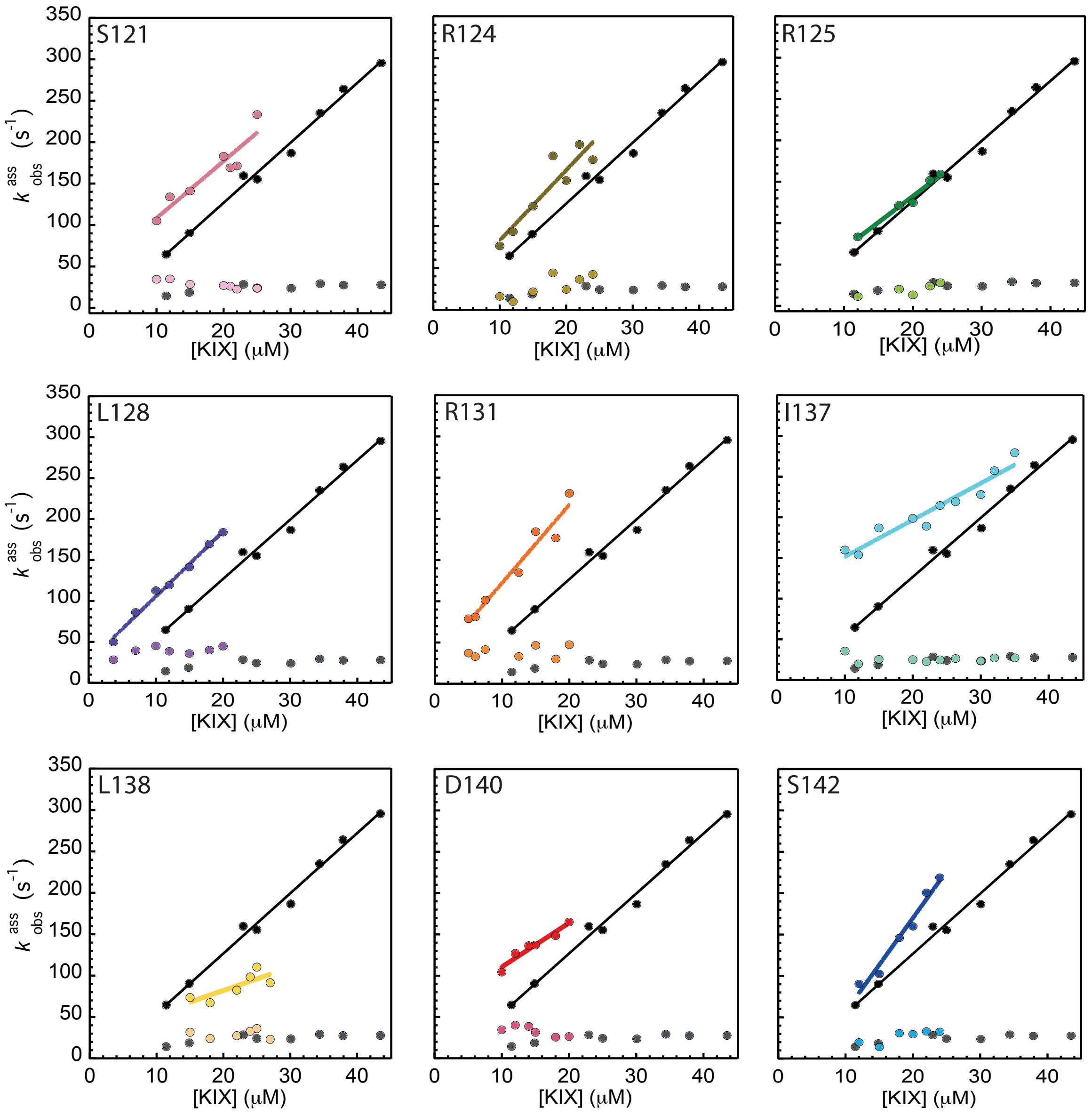

Figure S4A. Observed association kinetics rate of pKID and KIX for the interface mutations under pseudo-first order conditions with KIX in excess. Wild-type data is shown in black.

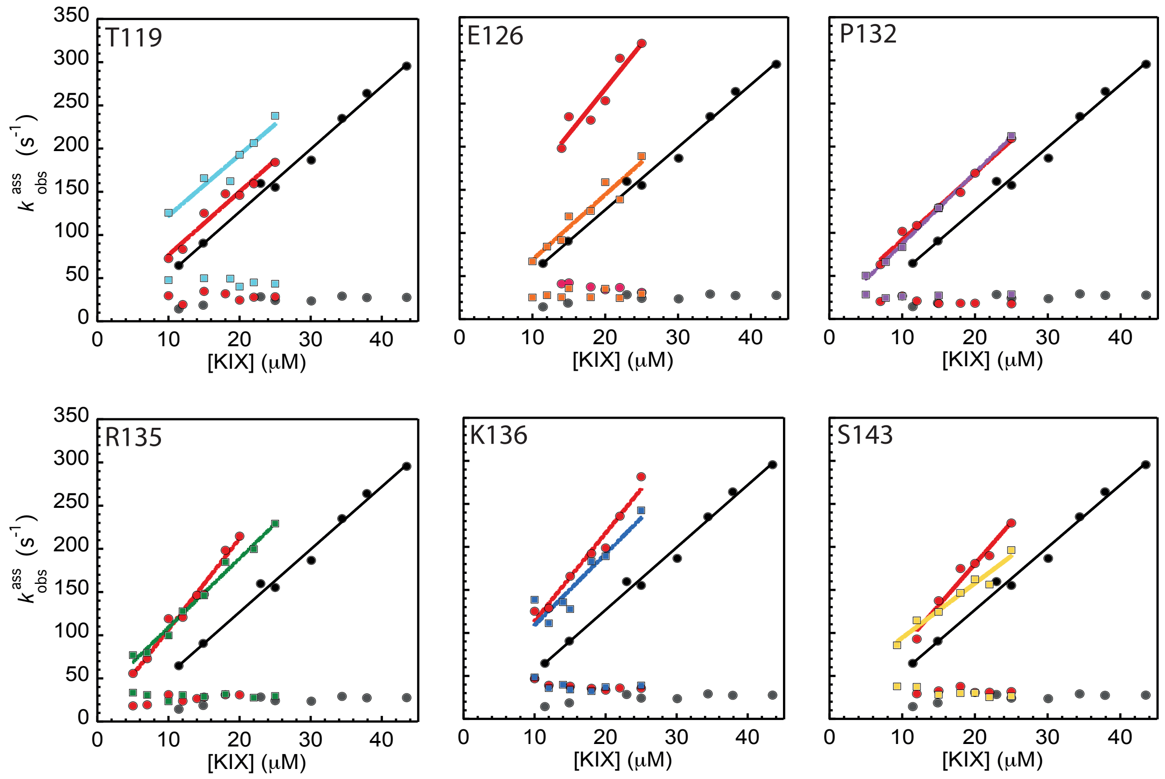

Figure S4B. Observed association kinetics rate of pKID and KIX for the solvent exposed pKID mutants obtained under pseudo-first order conditions with KIX in excess. Wild-type is shown in black and all Ala mutants are shown in red. Gly mutants are shown as labeled.

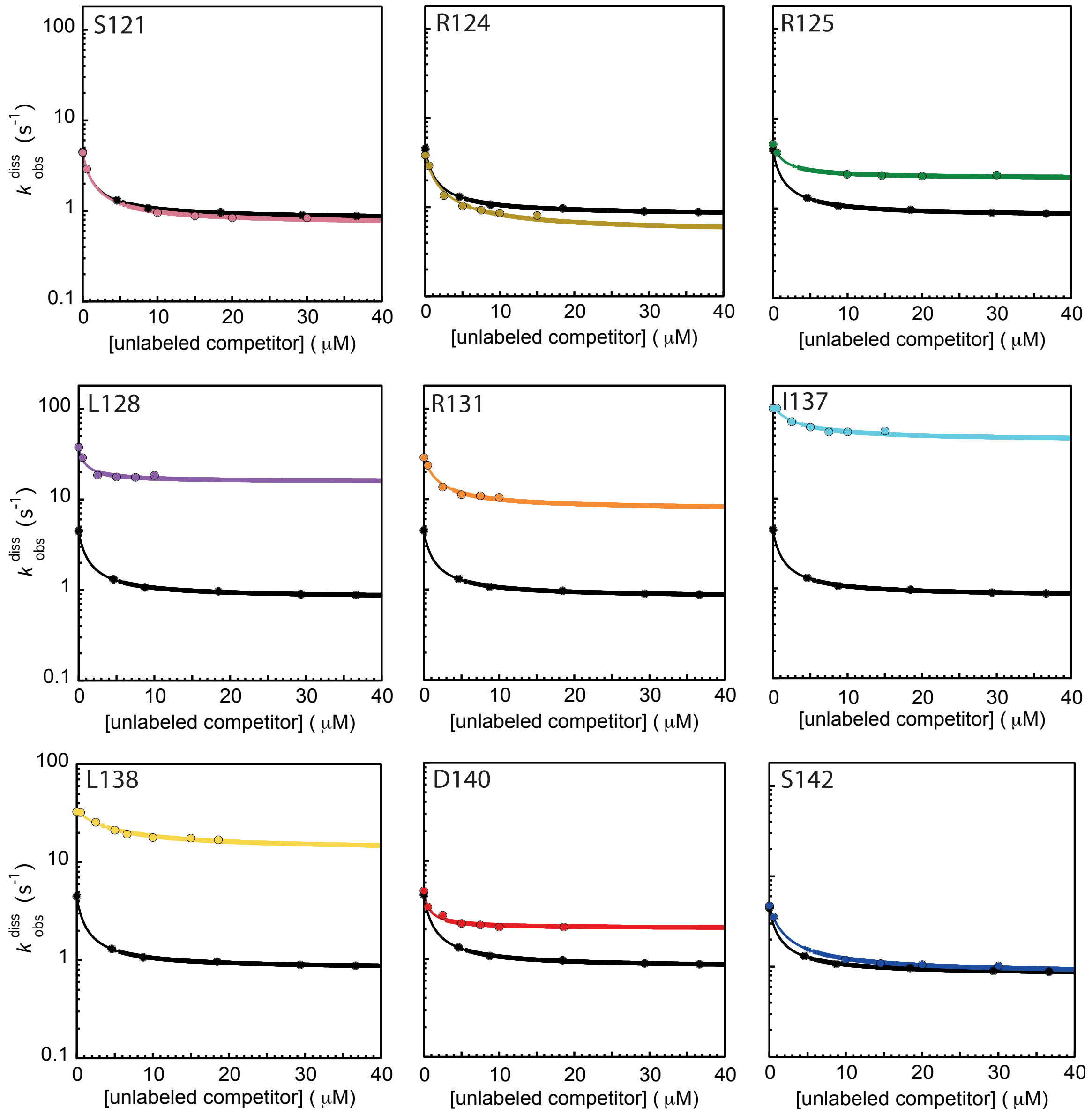

Figure S5A. Observed dissociation kinetics rate of wild-type (black) and interface pKID mutants from KIX obtained using cMybTAD as a competitor..

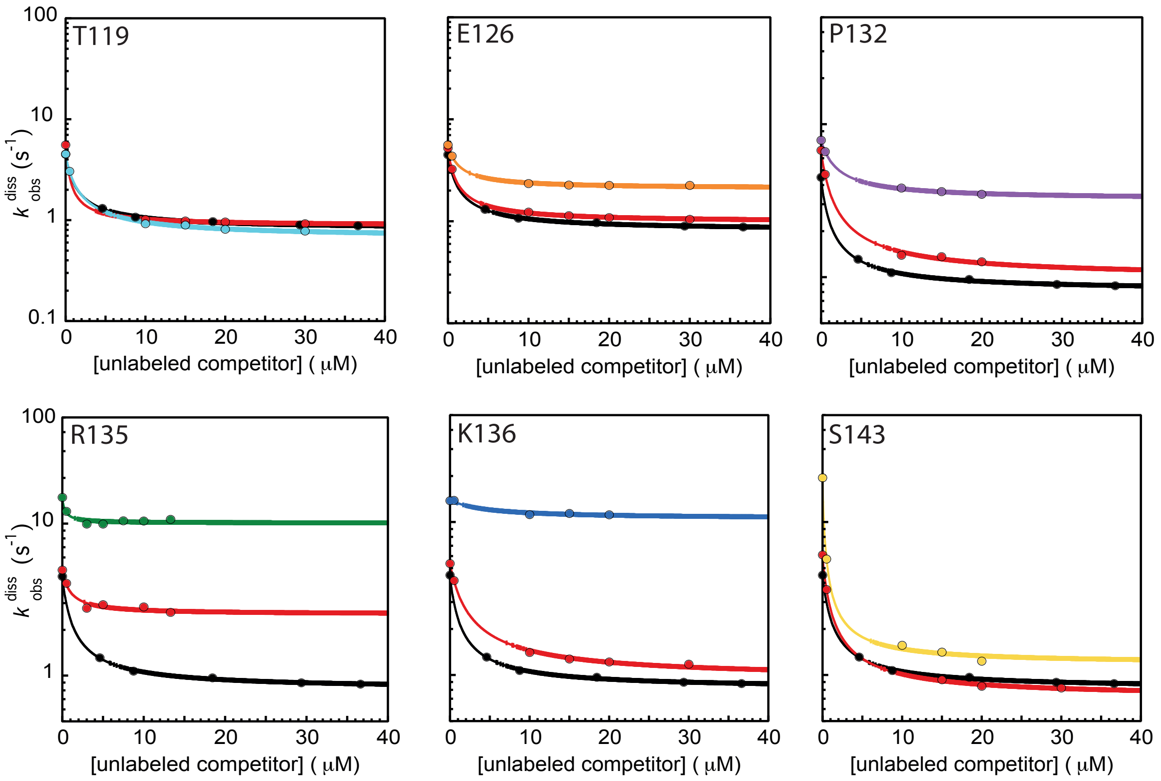

Figure S5B. Observed dissociation kinetics rate of wild-type (black spheres) and solvent exposed pKID mutants from KIX obtained using cMybTAD as a competitor. Wild-type is shown as black and all Ala mutants are shown in red. Gly mutants are shown as labeled.

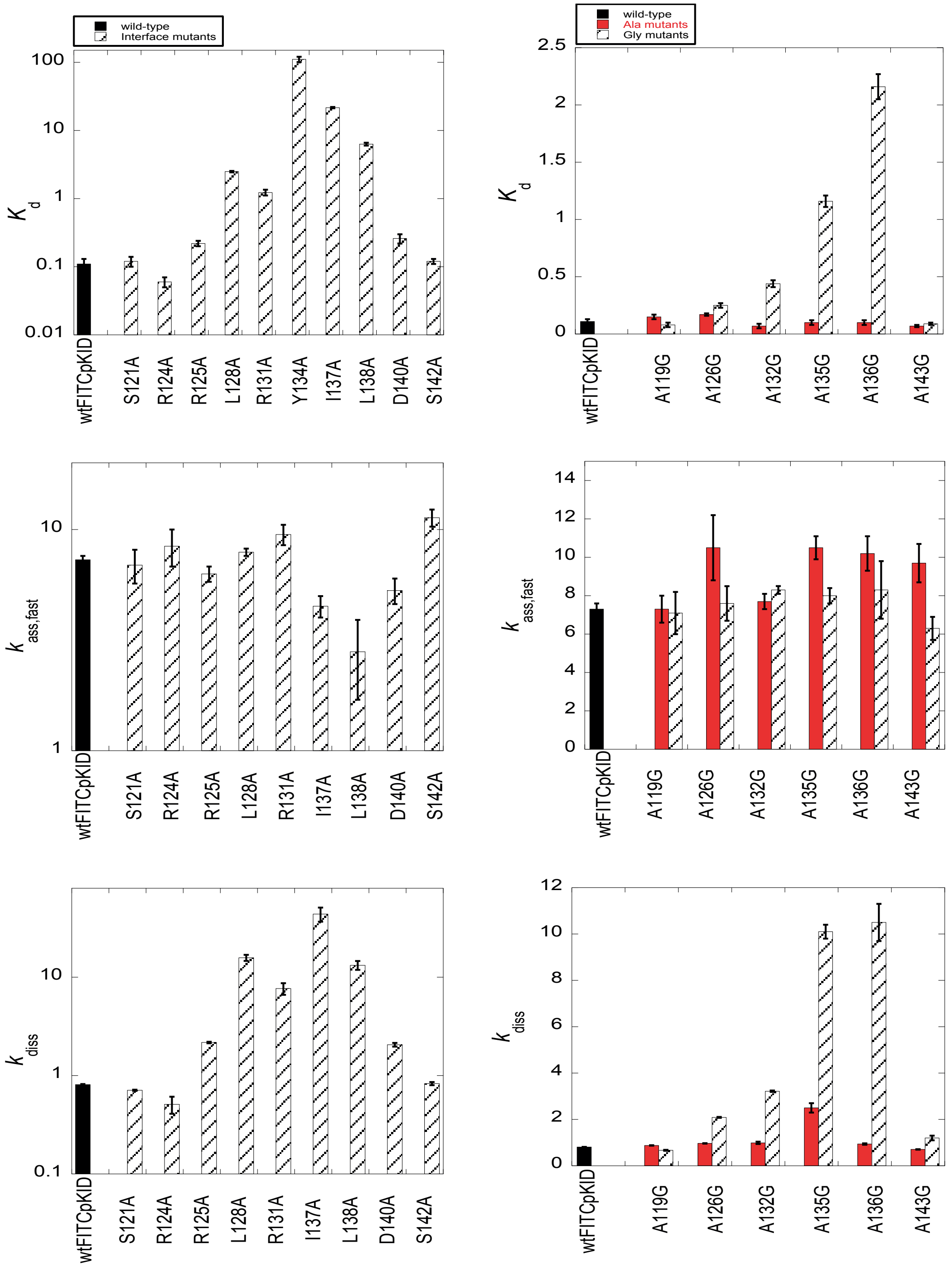

Figure S6. Bar chart showing equilibrium binding (K_d), fast association kinetics ($k_{ass,fast}$) and dissociation kinetics (k_{diss}) comparison of all mutants of pKID. Wild type is shown in filled black bar for all graphs. The bar charts on the left side show all interface mutants (hatched bar). The bar charts on the right show all Ala-Gly mutants. The Ala mutants are shown in red and the Gly mutants in hatched.

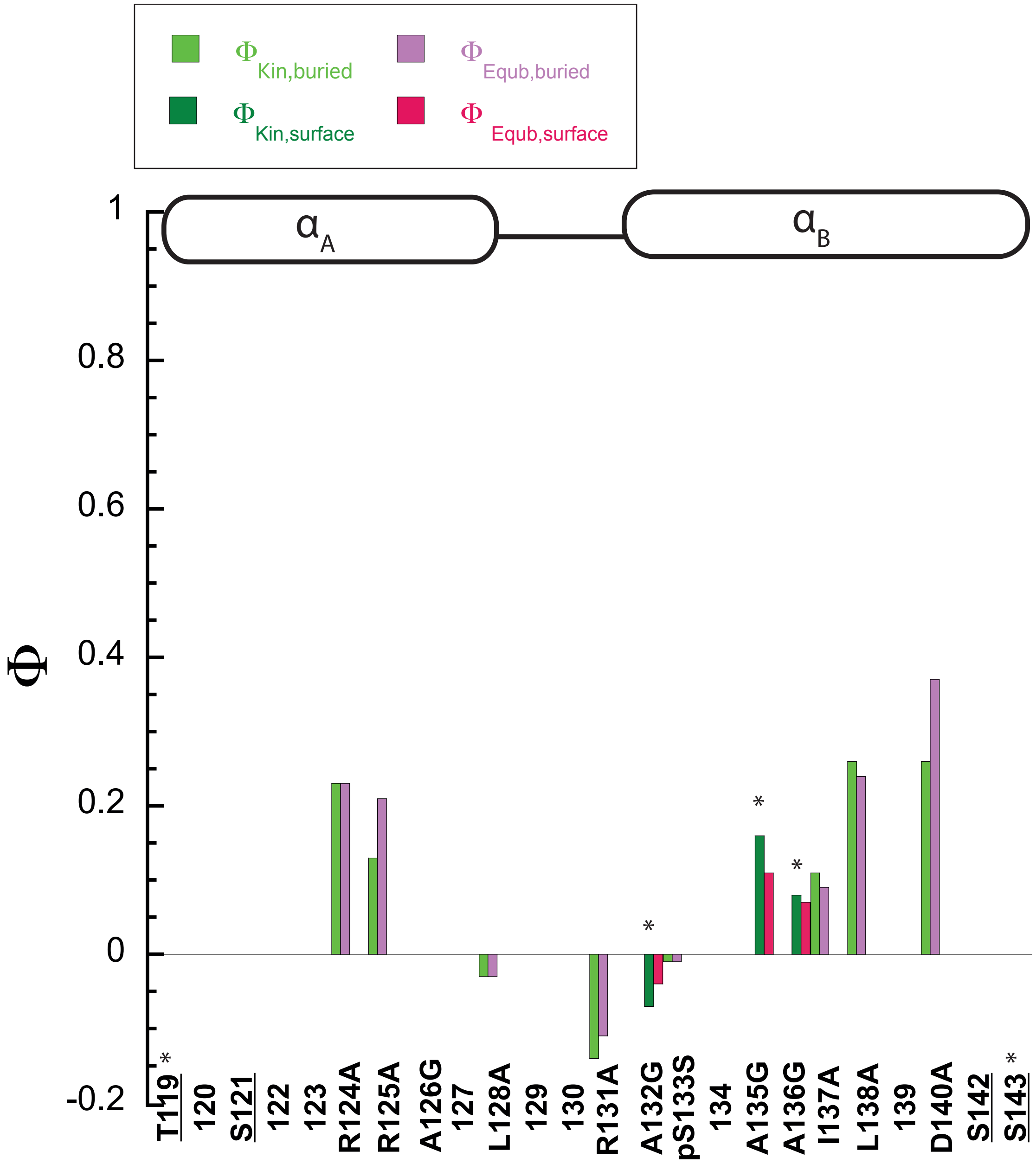

Figure S7. The pattern of Φ-values is the same whether using equilibrium or kinetic $\Delta\Delta G$. Bar chart showing comparison between Φ-values calculated using kinetic and equilibrium measurements. The Φ-values calculated using kinetic for interface mutants is shown in pale green and for surface mutants is shown in dark green. The Φ-values calculated using equilibrium for interface mutants is shown in pink and for surface mutants is shown in red. The * on top of the bar highlights the solvent exposed (Ala-Gly) mutations in the bar chart.
