# Supplementary material for: pKID Binds to KIX via an Unstructured Transition State with Nonnative Interactions
Source: Biophys J. 2017 Dec 19;113(12):2713–22. doi: 10.1016/j.bpj.2017.10.016 (PMC5770965; doi:10.1016/j.bpj.2017.10.016)
Supplement: Document S2. Article plus Supporting Material [file mmc2.pdf]

Biophysical Journal
Article

Biophysical Society

pKID Binds to KIX via an Unstructured Transition State with Nonnative Interactions

Liza Dahal,[1] Tristan O. C. Kwan,[1] Sarah L. Shammas,[1,*] and Jane Clarke[1,*]
[1]Department of Chemistry, University of Cambridge, Cambridge, United Kingdom

ABSTRACT Understanding the detailed mechanism of interaction of intrinsically disordered proteins with their partners is crucial to comprehend their functions in signaling and transcription. Through its interaction with KIX, the disordered pKID region of CREB protein is central in the transcription of cAMP responsive genes, including those involved in long-term memory. Numerous simulation studies have investigated these interactions. Combined with experimental results, these can provide valuable and comprehensive understanding of the mechanisms involved. Here, we probe the transition state of this interaction experimentally through analyzing the kinetic effect of mutating both interface and solvent exposed residues in pKID. We show that very few specific interactions between pKID and KIX are required in the initial binding process. Only a small number of weak interactions are formed at the transition state, including nonnative interactions, and most of the folding occurs after the initial binding event. These properties are consistent with computational results and also the majority of experimental studies of intrinsically disordered protein coupled folding and binding in other protein systems, suggesting that these may be common features.

INTRODUCTION

Intrinsically disordered proteins (IDPs) are central to protein interaction networks (1–3). Many of these IDPs undergo coupled folding and binding reactions, i.e., they fold to well-defined structures upon interaction with a partner protein (4–6). It has been argued that their disordered nature confers certain advantages over already folded proteins during protein-protein interactions (7–10). For example, enabling rapid binding and conformational changes when interacting with their partners (11–13), facilitating easy access to posttranslational modification (14,15), permitting alternative splicing and domain shuffles without perturbing structure of folded proteins (16,17), increasing plasticity, and allowing interaction with several binding partners (18,19). Many of these IDPs function at the hub of signaling and regulatory processes and are therefore abundant in eukaryotes (6,20–22). To date, most IDP studies involve computational or structural analysis and prediction of IDP ensembles, abundance or binding affinity with their partner (20,21,23–25). Fewer focus on understanding the mechanistic details of these interactions, which may be important in the search for "druggable" IDP targets (26).

Kinetic experiments, along with site-directed mutagenesis, have been used to perform Φ-value analysis to study transition states in protein-folding pathways (27–29). This method has been extended to studying the interaction between an IDP and its partner at residue level in a few studies (30–35). Comparable to protein-folding studies, association and dissociation kinetic rate constants and equilibrium constants can be used to calculate Φ-values. Traditionally, buried residues are shortened (e.g., to Ala) to probe the formation of the interface, and noninterface (solvent exposed) residues are mutated to Ala and then Gly to probe secondary structure (helix) formation. This allows us to probe the structure of the transition state of these IDP-partner interactions to understand the critical contacts formed during the coupled folding and binding pathway.

The Kinase Inducible Domain (KID) is an intrinsically disordered domain of CREB, which plays an important role in transcription regulation (36–39). On phosphorylation by Protein Kinase A (PKA), pKID binds to the KIX domain of CBP and folds into a kinked helical structure (40,41) (Fig.1). Phosphorylation increases the affinity of KID to KIX by increasing the lifetime of the complex (see the accompanying article by Dahal et al. in this issue of *Biophysical Journal*) (42). This lifetime is important because

Submitted July 12, 2017, and accepted for publication October 10, 2017.
*Correspondence: sarah.shammas@bioch.ox.ac.uk or jc162@cam.ac.uk
Sarah L. Shammas's present address is Department of Biochemistry, University of Oxford, Oxford, United Kingdom.
Editor: Francesca Marassi.
https://doi.org/10.1016/j.bpj.2017.10.016
© 2017 Biophysical Society.
This is an open access article under the CC BY license (http://creativecommons.org/licenses/by/4.0/).
CrossMark

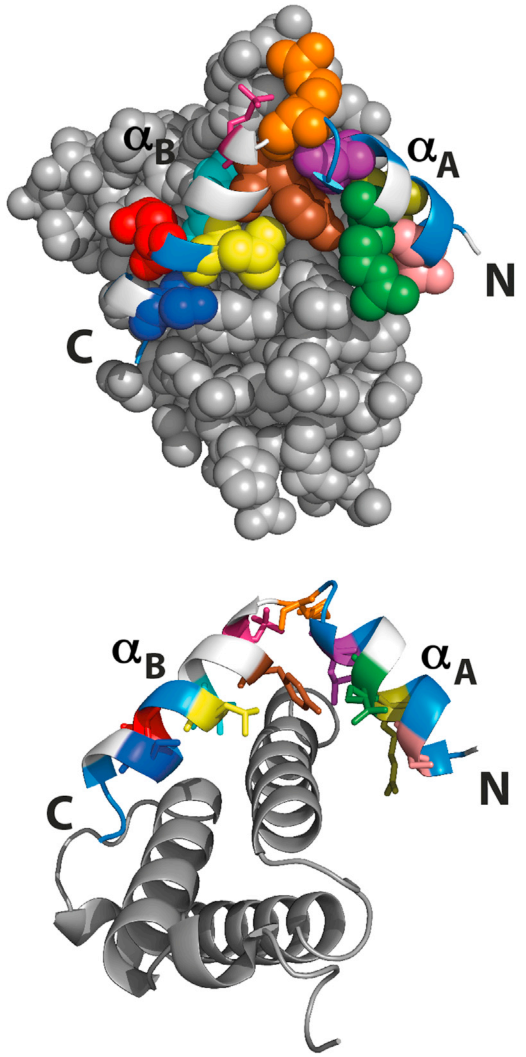

FIGURE 1 Structure of pKID (*cartoon*) and KIX (*gray spheres on top* and *gray cartoon on bottom*) showing interface and solvent-exposed residues of pKID that were mutated in the study. The phosphorylated serine of pKID is shown as sticks (*magenta*). The residues of pKID that contact the KIX interface are shown as spheres (*top*) and sticks (*bottom*); S121 (*pale pink*), R124 (*olive*), R125 (*green*), L128 (*purple*), R131 (*orange*), Y134 (*brown*), I137 (*cyan*), L138 (*yellow*), D140 (*red*), and S142 (*dark blue*). The mutated solvent-exposed residues of pKID are shown in white.

it will set a timescale for recruitment of transcription factors to initiate transcription. The crucial interaction between pKID and KIX is therefore of interest, and it is important to understand the mechanistic details of this interaction. The pKID-KIX system has previously been studied using NMR and computational studies (43–50). These studies suggest that pKID binds to KIX via an induced fit mechanism. A partly structured intermediate is detected in equilibrium NMR studies (43). Here, we use Φ−value analysis to provide an insight into the early rate-limiting transition state for the pKID-KIX interaction. We find that the transition state for assembly/disassembly is mostly unstructured, as suggested by previous studies (45–47). Interestingly, we find that the extreme N- and C-terminal regions of KID are partly structured and packed at the transition state, but that in the interhelical kinked region, which includes the phosphorylation site, there is no evidence for native structure formation; rather, the data suggest that this region may form nonnative contacts at the transition state.

MATERIALS AND METHODS

Expression and purification of KIX was carried out as described previously (51). N-terminal labeled wild-type and mutants of FITC-pKID peptides (UniProt P15337, residues 116–146) were purchased from Biomatik (Ontario, Canada). Dilutions using biophysical buffer and concentration determinations, and all biophysical experiments were carried out as described in the accompanying article (42).

Φ-value calculations

Φ-values were calculated using both equilibrium and kinetic data using the following equations:

$$\Phi_{\mathrm{Equb}} = \frac{\ln\left(k_{\mathrm{ass,fast}}^{\mathrm{wild-type}} \middle/ k_{\mathrm{ass,fast}}^{\mathrm{mut}}\right)}{\ln\left(K_{d\ \mathrm{Equb}}^{\mathrm{mut}} \middle/ K_{d\ \mathrm{Equb}}^{\mathrm{wild-type}}\right)} \quad (1)$$

$$\Phi_{\mathrm{Kin}} = \frac{\ln\left(k_{\mathrm{ass,fast}}^{\mathrm{wild-type}} \middle/ k_{\mathrm{ass,fast}}^{\mathrm{mut}}\right)}{\ln\left(K_{\mathrm{d\ Kin}}^{\mathrm{mut}} / K_{\mathrm{d\ Kin}}^{\mathrm{wild-type}}\right)}. \quad (2)$$

The association ($k_{\mathrm{ass,fast}}$) and dissociation (k_{diss}) rate constants were used to calculate the kinetic K_{d}:

$$K_{\mathrm{d\ Kin}} = \frac{k_{\mathrm{diss}}}{k_{\mathrm{ass,fast}}}\ , \quad (3)$$

where $k_{\mathrm{ass,fast}}$ represents the fast association rate constant, obtained from the gradient of the straight line used to fit the observed fast association rate at different KIX concentrations. k_{diss} represents the dissociation rate constant, obtained from the asymptote of the plot used to fit the observed apparent dissociation rate constants of wild-type and mutant pKID from KIX at different, unlabeled competitor concentrations.

Errors of the fit in equilibrium constant and association and dissociation rate constants were used for the error calculations. Errors in Φ-values were calculated using SE propagation methods.

RESULTS

Selection of pKID mutants

N-terminal, FITC-labeled pKID peptides are used for all the experiments reported here.

To perform the Φ-value analysis, we introduced mutations to probe inter- and intramolecular contacts in the pKID-KIX system. Interface mutants, residues in pKID that come in contact with KIX (E. Eyal et al., 2009, Weizmann. Inst. Sci., conference), were mutated to Ala to interrogate the contacts made at the peptide-protein interface (Fig. 1). Ala-Gly scanning is an established method used

to probe formation of helical secondary structure (52–54). Exposed (i.e., noncontacting) side chains were first mutated to Ala and then to Gly. Substitution of the Cβ, which only makes intrahelical contacts by Gly (known to disfavor helix formation), provides a specific probe of secondary (helical) structure formation in the transition state. Ala-Gly scanning mutations (six solvent exposed residues of pKID, three in each helix) were introduced to probe helix formation (secondary structure formation) (Fig. 1).

Effect of pKID mutations on residual helicity

All biophysical data are included in the supplementary information. In Fig. 2, we show biophysical data for three representative variants: one from N-terminal helix-A (α_A), one from the interhelical kinked region, and one from C-terminal helix-B (α_B). We use circular dichroism measurements to estimate how the mutations of pKID affect its residual helicity (Fig. S1). As reported in the accompanying article, the residual helicity of pKID in absence of KIX is around 17% (42). Overall, the effect of mutation on the residual helicity was small (Fig. S2). In comparison to the wild-type, Ala mutations at the protein-protein interface of pKID-KIX either decrease or have no effect on residual helicity. Mutation of solvent exposed residues to Ala had little to no effect on the residual helicity. As expected, Gly mutations slightly reduce helicity of the pKID peptides (except for peptide S143G where the helicity is similar to wild-type).

Effect of pKID mutations on the stability of the complex

Equilibrium binding constants were determined using fluorescence anisotropy (Fig. S3). The more destabilizing mutations are near the interhelical kinked region and toward the C-terminus of pKID (Tables S1 and S2). Y134A increases K_d by three orders of magnitude, I137A by two orders of magnitude, and L138A and L128A over 10-fold (Tables S1 and S2). Of these Y134, I137, and L138 have previously been described as forming the hydrophobic motif ΦXX$\Phi\Phi$ (where Φ is a bulky hydrophobic residue) for KIX binding. Of the solvent-exposed mutants, the Gly mutants in α_B, closest to the interhelical kinked region of pKID (A132G A135G, A136G), are more destabilized than others. Ala mutants in this case have either a similar or lower K_d than wild-type. $\Delta\Delta G$ values reported in Table S2 (surface mutations) are for the composite Ala-Gly mutations and are generally lower than $\Delta\Delta G$ for interface mutations.

Effect on association and dissociation rates

Association kinetics experiments for all mutants show two phases. With the pseudo-first-order conditions and concentration range we have investigated, we observe a fast rate ($k_{ass,fast}$), which appears to be linearly dependent on concentration, and a slow rate ($k_{ass,slow}$) that appears to show little or no concentration dependence. We discuss these fast and slow rates for wild-type FITC-labeled pKID in the accompanying article (42). Neither rate varied significantly upon mutation (Fig. S4), with the most notable change of the fast association rate constant ($k_{ass,fast}$) being only ~1.5-fold. It was not possible to obtain a signal change for Y134A (a highly destabilizing mutation), most likely because the K_d was too high to significantly populate the bound complex and thus allow an observable change in fluorescence in the stopped-flow experiments. In marked contrast, we observe significant changes in the observed dissociation rate constant (k_{diss}) upon mutation (Fig. S5). Thus, the change in stability for both the interface and solvent-exposed mutants is due almost entirely to changes in k_{diss} (Fig. 3; Fig. S6).

Using Φ-value analysis to probe the structure of the transition state

Φ-values for both interface and solvent-exposed mutants were calculated using dissociation constants determined using both the equilibrium and kinetic data (K_{dEqub}, obtained from equilibrium binding experiments and $K_{dKin} = k_{diss}/k_{ass,fast}$). We see the same pattern of Φ-values using both methods of calculation (Fig. S7). Previous examples in the literature have only used mutants with sufficiently large $\Delta\Delta G_{D\text{-}N}$ to reliably calculate Φ-values (31,34,35). Thus, we only report Φ-values where both equilibrium and kinetic $\Delta\Delta G_{D\text{-}N} \geq 0.34$ kcal mol^{-1} (Tables S1 and S2). Note that the surface Ala-Gly mutations were generally not sufficiently destabilizing to allow Φ-values to be determined. In total, 11 Φ-values could be calculated. Overall, the Φ-values for both secondary structure probing Ala-to-Gly mutations and for interface mutations are low, with the highest Φ-values around 0.3–0.4, suggesting that at the transition state, no region of pKID is either fully structured or fully bound.

DISCUSSION

Most residues in α_A are either charged or polar, and as has been shown in the previous NMR structural studies, make minimal contact with KIX. Thus, as has been observed previously (47,55), the $\Delta\Delta G$ values for interface mutations in this helix are generally low compared with similar mutations in α_B. Previous structural studies show that side chains of Tyr134, Ile137, Leu138, and Leu141 in pKID interact with a shallow hydrophobic groove of KIX (helices A and B), which forms one of the two main binding sites in KIX (40; E. Eyal et al., 2009, Weizmann. Inst. Sci., conference). The most destabilizing mutations are those that delete most contacts between the peptide and KIX in this groove (48). R131A also causes significant complex destabilization,

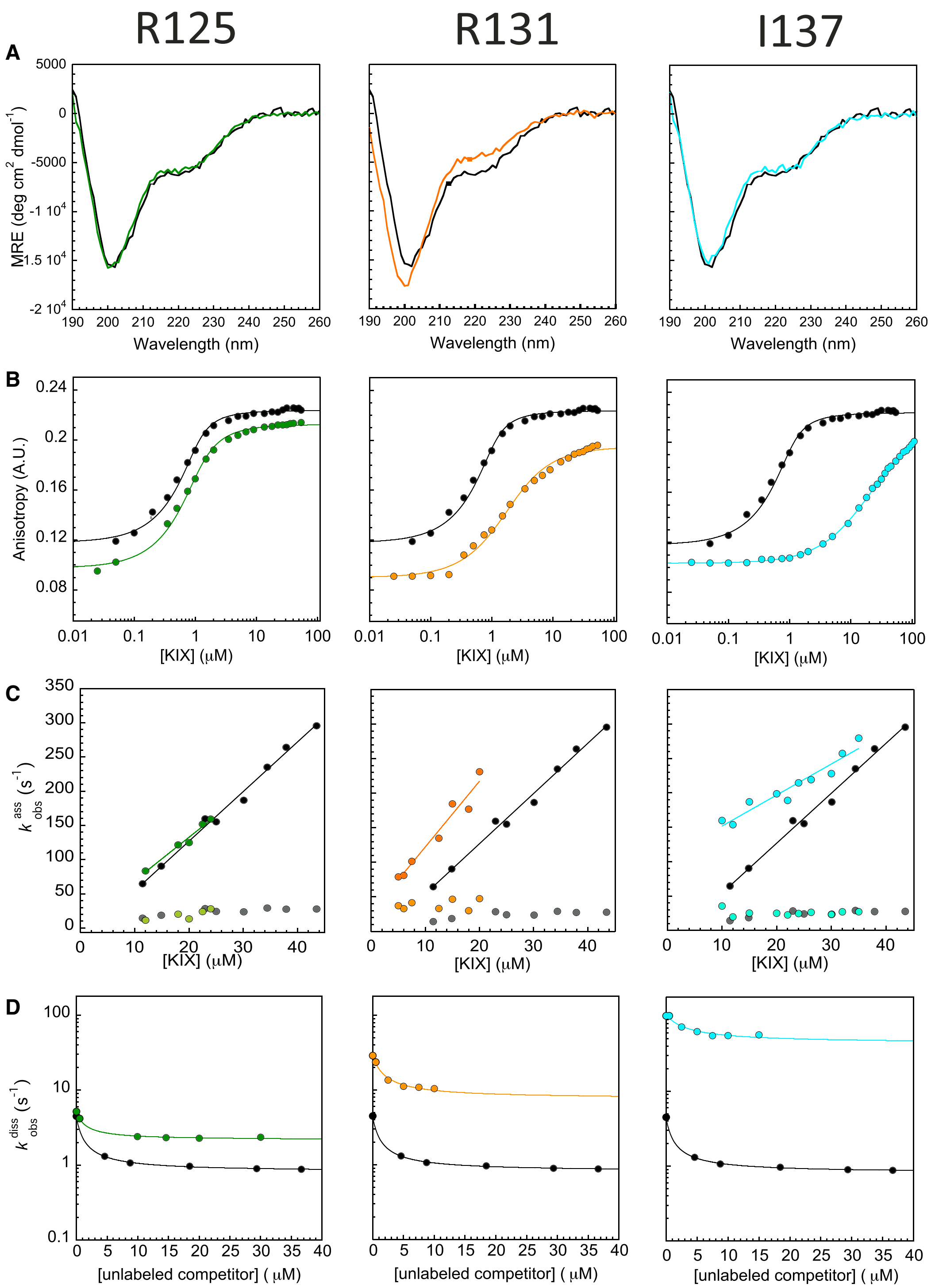

FIGURE 2 Sample biophysical data. (*A*) Circular dichroism (CD), (*B*) equilibrium anisotropy, and (*C*) association and (*D*) dissociation kinetic plots for mutants of three different residues along the length of pKID. Wild-type data are shown in black/gray for comparison. The left panels show data for R125A (*green*) in α_A of pKID. The middle panels show data for R131A (*orange*) in the interhelical kink region. The right panels show data for I137A (*cyan*) in α_B of pKID.

probably because its interaction with the phosphate group is important for KIX-pKID interaction (56). Moreover, NMR and simulations agree that α_A is largely helical in the unbound state (43,47). Thus, Ala-to-Gly mutations destabilize both the unbound pKID and the bound state, so none of the surface mutations were sufficiently destabilizing to allow Φ-values to be determined for surface residues in α_A. In contrast, in α_B, which is largely unstructured in the unbound form, Ala-to-Gly mutations were found to be far more destabilizing.

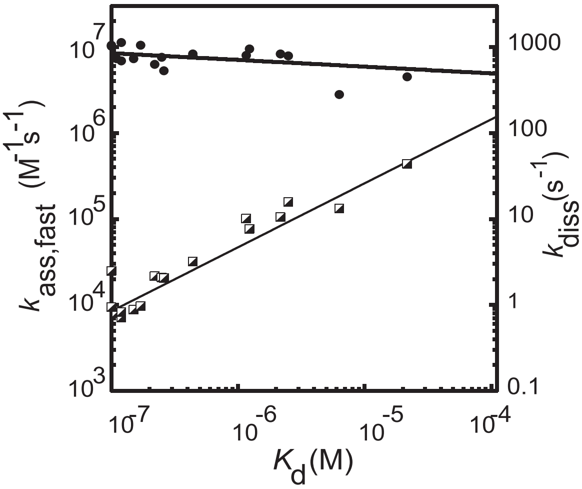

FIGURE 3 Linear free-energy plot. $k_{ass,fast}$ (●) and k_{diss} (◩) plotted against K_d for all mutants of pKID binding to KIX. Changes in K_d are mostly due to changes in k_{diss}.

Observation of a slow rate in the association kinetic experiments

For all mutants investigated we observed biphasic association kinetics, as previously reported for FITC-labeled wild-type KID and pKID (42,44). Under our conditions, the fast phase ($k_{ass,fast}$) is linearly dependent upon protein concentration; however, the second, slower phase ($k_{ass,slow}$) has lower amplitude and appears concentration independent. Although we cannot assign the origin of the second rate (see accompanying article for discussion), we have determined that it represents a unimolecular transition that takes place after the initial association reaction (42). The slow rate appears to be relatively insensitive to mutation, differing only for a few mutants by a maximum of ~1.5-fold (Fig S4). Furthermore, there is no systematic pattern for the few residues that do apparently have a different $k_{ass,slow}$ (e.g., T119G, but not T119A, shows increased $k_{ass,slow}$, as does K136, but not adjacent I137). The presence of this extra phase does not prevent us using Φ-value analysis to investigate the first rate-determining transition state for the association reaction, as we discuss next.

Comparing K_d and $\Delta\Delta G$ obtained from equilibrium and kinetic experiments

The equilibrium binding constant K_d, is related to the free energy difference between initial and final states of a reaction, and rate constants are related to the free energy difference between the initial/final state and the transition states. For a two-state reaction, K_d can be determined from the ratio k_{off}/k_{on}, and this will match the value determined from equilibrium experiments. For three-state reactions with two populated bound states, as appears to be the case here, the equilibrium and kinetic K_d values do not necessarily match each other, although they can be almost identical depending upon the relative values of the various rates. In the accompanying article we showed that the estimates of K_d from kinetic experiments (using $K_{dKin} = k_{diss}/k_{ass,fast}$) and equilibrium experiments are the same within error for the wild-type FITC-pKID-KIX association reaction (42). When we compare the K_{dEqub} with those obtained from kinetics for all the pKID mutants investigated in our study, we also see a generally good agreement. Consequently, the changes in free energy of binding upon association ($\Delta\Delta G$) are also in agreement (Fig. 4). Thus it appears that for FITC-pKID, two of the observed rates, k_{diss} and $k_{ass,fast}$, are sufficient to reflect the free energy change between unbound pKID and KIX bound forms.

K_{dEqub} reflects the difference in free energy between the unbound and bound ensembles. The bound ensemble

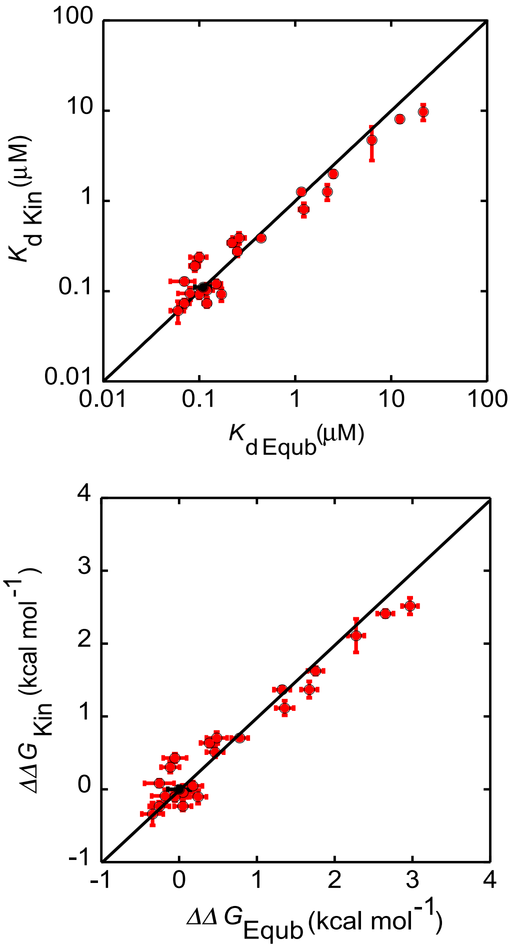

FIGURE 4 Comparison between kinetic and equilibrium K_d and $\Delta\Delta G$. (*Top plot*) K_{dKin} plotted against K_{dEqub} for all pKID mutants. (*Bottom plot*) $\Delta\Delta G_{Kin}$ plotted against $\Delta\Delta G_{Equb}$ calculated for all pKID mutants. The straight line of $y = x$ shown in black is presented to guide the eye for comparison between kinetic and equilibrium measurements. Wild-type parameters are shown in black and all other mutants are shown in red. Where affinities are lower this results in more uncertainty in the K_d measurements, and thus larger differences between kinetic and equilibrium data.

includes both "intermediate" and "final" forms, and it is well defined because at equilibrium, the bound species are always present in a constant ratio to each other. We are therefore able to perform Φ-value analysis by comparing the changes in $k_{ass,fast}$ and K_{dEqub} upon mutation, to probe structure formation in the first transition state (compared with that in the bound ensemble). Here we used both the kinetic and equilibrium $\Delta\Delta G$ values to calculate the Φ-value. Similar analysis has been used previously for Φ-value analysis of ACTR-NCBD, where a second phase was observed in association kinetics in the presence of TMAO (34). Importantly, our Φ-value analysis reports on the transition state of the initial binding interaction of pKID with KIX.

Φ-value analysis indicates very little structure formation at the transition state

We were able to determine only 11 Φ-values: three in α_A (all interface), five in α_B (three interface, two surface), and three in the interhelical kinked region. We note that we have no Φ-values in the first half of α_A or toward the end of α_B. Because the errors in Φ are relatively high (Tables S1 and S2), we cannot make any statement about specific individual interactions that are crucial in early structure formation. However, the pattern of Φ-values is consistent so here we interpret only this pattern of Φ-values, which is described in Fig. 5. The most important observation is that all Φ-values are low, suggesting that the rate-limiting transition state for formation of the initial complex is largely unstructured.

We see evidence for weak, but early contact formation in the central part of α_A, although we have only two Φ-values here, residues R124 and R125, both of which form direct contacts with KIX in the complex. Because both are positively charged, it is unlikely that the observed slowing of association comes from global electrostatic effects; pKID becomes more negatively charged on mutation of R124 or R125 to Ala, which would be predicted to speed association. Note that we do not report Φ-values for E126A in Fig. 5 because the $\Delta\Delta G$ values from equilibrium and kinetic data do not agree, although the Φ-values calculated are positive (Table S2). We have no information about the N-terminal region of this helix, nor can we say that contact formation is concomitant with helical structure formation. The C-terminal residue of this helix, L128, which is buried in the KIX interface, has a Φ-value close to zero. Thus our data simply suggest that some contacts between residues in this central region of the α_A of pKID and KIX are formed early.

Interestingly, we see more evidence for early association of the α_B helix, particularly in its central region. Residues in α_B form more native contacts with KIX (Fig. 5; Tables S1 and S2), particularly residues I137 and L138 that are among the most destabilizing mutations. Y134, I137, L138, and L141 are all known to interact with the primary docking

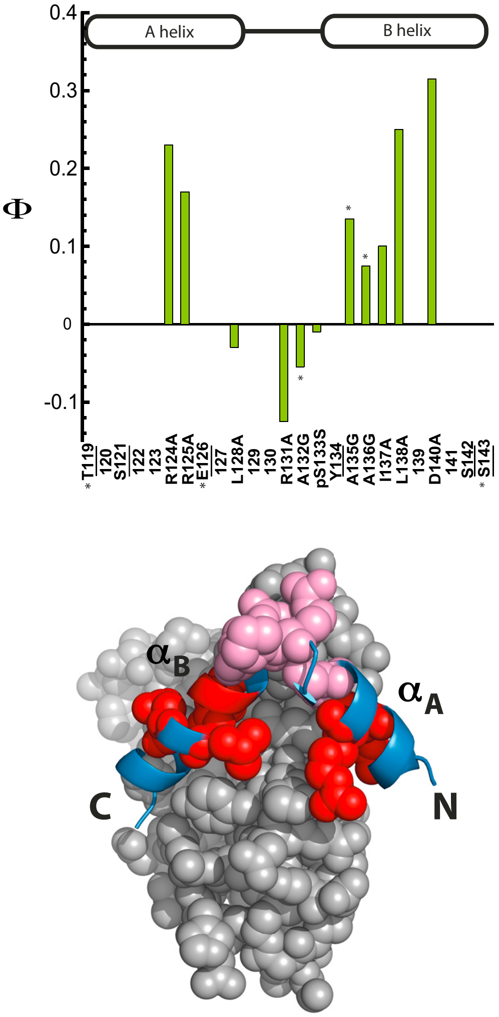

FIGURE 5 Φ-values for interface and solvent-exposed mutants. Top: $\Phi_{Average}$, average of Φ-values calculated using both kinetic and equilibrium methods are plotted for mutants with $\Delta\Delta G > 0.34$ kcal mol^{-1}. The residues that were investigated but where Φ-values could not be calculated are underlined. Solvent-exposed (Ala-Gly) mutants are highlighted by an asterisk. Bottom: the pattern of Φ-values is mapped on the structure of pKID/KIX (1KDX). Φ-values are generally low, so negative Φ-values are shown in pale pink and positive Φ-values are shown in red.

groove of KIX (40), but we were only able to determine Φ-values for two of these residues. We were unable to obtain kinetic data for Y134A, and the company that provided us with the peptides was unable to synthesize L141A. The two remaining interface residues, L138 and D140, have the highest Φ-values in our studies. Although we could not probe the C-terminal part of α_B, both surface and buried residues at the N-terminal part of this helix have positive Φ-values. Thus we have evidence for extensive, albeit only weak, structure and contact formation between α_B and KIX at the transition state.

Perhaps the most striking result, however, is the pattern of negative Φ-values shown by residues in the interhelical kink region (Fig. 5): this includes three interface residues that

make contact with KIX (L128, R131, pSer133) and one surface residue (P132). We note that the interface residues are very different in character: L128A deletes hydrophobic interactions, R131A deletes both hydrophobic packing and a positive charge, and pSer to Ser removes negative charges. A negative Φ-value indicates that removal of these interactions stabilizes the transition state: in all these cases the mutation actually enhances the rate of formation of the transition state, albeit marginally. This result suggests that residues in the interhelical region may be forming nonnative interactions in the transition state. Such nonnative interactions have been inferred from simulation experiments (45,50). Contrary to previous proposals (56,57) the low or negative Φ-values in this region suggest that phosphorylation does not play a role in initiating the binding of pKID to KIX (42).

Comparison with previous studies

The pKID-KIX interaction is a paradigm in folding upon binding and thus has been the focus of a number of simulation and experimental studies (40,43,45,47–50,55,58). Here we compare our results with these studies. Sugase et al. (43) investigated pKID-KIX assembly using a variety of NMR techniques. They observed formation of transient interactions between pKID and KIX and suggest that pKID can bind nonspecifically to a number of sites on KIX. They ascribe these observations to formation of an ensemble of structures that comprise the early encounter complex and suggest that this is dominated by formation of hydrophobic interactions, in particular between a partly formed α_B pKID and KIX. They propose that this complex then evolves (i.e., via an induced fit mechanism), by a diffusive process and without dissociation, to form the bound state.

There are also three detailed simulation studies of the mechanism of pKID folding upon binding to KIX; these are in remarkable agreement with each other, although they use different methodologies (47,49,50). In all cases the unbound KID structure reflects that seen in NMR studies (58), that is, α_A has significant residual helical structure, whereas α_B is essentially unfolded. The initial encounter complex which leads to productive folding is always observed to be almost as unfolded as pKID alone, and in all cases this is dominated by interactions between α_B (in particular between the C-terminal region of α_B) and KIX (47,49). Furthermore, nonnative interactions between other regions of pKID and KIX are also detected in all the simulations. In general, these nonnative interactions are neither specific, nor long-lived, although Umezawa et al. (59) detect binding to a specific, alternative (MLL) binding site in KIX.

Our data are consistent with all these studies. All Φ-values are low, indicating that pKID is not significantly more structured than in the unbound state. We observe positive Φ-values for two surface Ala-to-Gly mutations in the start of α_B, but the interface Φ-values are generally higher, suggesting that α_B is indeed packing onto KIX before folding into a helix. The C-terminal region of this helix has higher Φ-values than the N-terminal end (with residues L138 and D140 having the highest overall Φ-values), as suggested by the simulations (47). Huang and Liu (45) investigated the role of nonnative interactions of pKID-KIX binding in detail in their simulation studies. They point out that nonnative interactions can both speed and slow association; the patch of negative Φ-values we detect in the interhelical loop would indicate that the nonnative binding of this region mainly speeds association (45).

There is an alternative explanation for our observation of the interactions of α_A. One major difference between the NMR and simulation studies is how folding upon binding proceeds from the encounter complex (43,47,49). In all cases an intermediate (or intermediates) is detected, but in all simulations studies one sees consolidation of binding and folding of the α_B before α_A binds. Indeed, this is what our data lead us to infer as being the likeliest scenario. We detect apparently significant interactions between α_B and KIX at the transition state and, because interactions between α_B and KIX provide most of the interaction energy (40,41,48), it seems unlikely that α_B would unbind after the rate-limiting transition state, with consolidation appearing more likely.

In the NMR studies an intermediate with α_A folded and α_B largely unfolded and detached was inferred. Interestingly, in one of the simulations (47) a low occupancy alternative folding pathway is detected, whereby α_A folds and binds before α_B. Ganguly and Chen (49) also observed a similar, low frequency, early formation of an α_A-folded intermediate, but in their simulations, this is off-pathway and does not lead to productive folding. We cannot rule out the possibility that the two positive Φ-values we observe in α_A reflect this alternative route. However, it is important to note that both these simulations studies (47,49) can reconcile their observation of the dominant, α_B-first pathway with the interpretation of the NMR kinetic data from Sugase et al. (43). Unfortunately, as we are unable to probe folding of the intermediate in our kinetic studies, we are unable to shed further light on this controversy.

CONCLUSIONS

NMR and simulation techniques have been used to study the pKID-KIX interaction extensively (40,43,47–50). Our results add experimental evidence using kinetic and thermodynamic techniques to probe the mechanism of assembly in more detail. In the accompanying article (42) we demonstrated that the kinetic signature of this reaction is predominantly that of an induced fit mechanism, consistent with previous studies (43,47,60,61); that is, that folding occurs after binding, and therefore no particular conformation in the disordered ensemble is required for binding to occur. Our Φ-value analysis presented here is consistent with

this; pKID forms very little secondary structure or interface contacts with KIX at the transition state. pKID apparently requires only a few native interactions between α_B and KIX to commit to complex formation, whereas other regions of the peptide appear to play a role in formation of weak, possibly nonnative contacts. This suggests that the transition state may resemble the so-called "fuzzy" complexes that are the final bound state of some IDPs (62,63), probably best described by Turjanski et al. (47) as "a broadly distributed ensemble of conformations in which pKID binds to KIX in different conformations," but with interactions made by α_B being key to complex formation. Interestingly, both the NMR and simulation studies also suggest that the final bound complex of pKID-KIX is itself highly mobile, with α_A in particular being only loosely bound.

When considering mechanisms of folding upon binding it is important to bear in mind that different mechanisms may be relevant under different conditions; parallel modes of folding upon binding are likely to exist in all systems (30,32–35,64). For example, high concentrations will favor conformation selection over induced fit for kinetic reasons (65,66). Currently, the majority of Φ-value analyses (30,31,34,35,67,68) and simulation studies (69–71) also observe a relatively unstructured transition state for the interaction of IDPs with their partners, and most propose a (largely) induced fit mechanism where the majority of folding occurs after the rate-limiting binding step.

Blackledge and co-workers have proposed that folding and binding is better described by mixed mechanisms (72), perhaps where transient residual structure in a small section of the IDP plays a key role in binding. Computer simulations and equilibrium NMR studies have suggested a conformation selection mechanism for the interaction of intrinsically disordered C-terminal domain of the measles virus nucleoprotein and X domain of the viral phosphoprotein (73,74) and c-myb-KIX (75,76). However, it is important to recognize that mere existence of residual structure does not of itself mean that this is the region of the protein that binds first (26,77,78). Recent kinetic studies on some of these systems propose that binding occurs before folding (79,80).

So far, the focus has largely been on disordered proteins that fold into very simple topologies upon binding, which could introduce a potential bias. Where both partners are disordered before assembly, the reaction may be more complex. A mixed conformational selection plus induced fit mechanism has been proposed for both the assembly of the spectrin tetramerization domain, where association is slow and significant structure is formed at the transition state (33) and for the interaction between ACTR and NCBD (34,81,82). For the ACTR and NCBD system, association is fast and the transition state relatively disordered, but folding to the final structure is slow. In general, so far it appears that assembly reactions characterized by relatively unstructured transition states, such as that we observe here between pKID and KIX, may be a general theme for allowing fast, coupled folding and binding reactions.

SUPPORTING MATERIAL

Seven figures and two tables are available at http://www.biophysj.org/biophysj/supplemental/S0006-3495(17)31131-1.

AUTHOR CONTRIBUTIONS

L.D., S.L.S., and J.C. designed research and analyzed data. L.D. performed research. T.O.C.K. expressed and purified proteins. L.D., S.L.S., and J.C. wrote the manuscript.

ACKNOWLEDGMENTS

The authors thank Annette Steward for reading and providing comments on the manuscript.

This work was supported by the Wellcome Trust (grant WT095195). J.C. is a Wellcome Trust Senior Research Fellow. S.L.S. is a Medical Research Council Career Development Fellow (award MR/N024168/1). L.D. is supported by an Engineering and Physical Sciences Research Council (UK) studentship.

REFERENCES

1. Patil, A., K. Kinoshita, and H. Nakamura. 2010. Domain distribution and intrinsic disorder in hubs in the human protein-protein interaction network. *Protein Sci.* 19:1461–1468.
2. Kim, P. M., A. Sboner, ..., M. Gerstein. 2008. The role of disorder in interaction networks: a structural analysis. *Mol. Syst. Biol.* 4:179.
3. Haynes, C., C. J. Oldfield, ..., L. M. Iakoucheva. 2006. Intrinsic disorder is a common feature of hub proteins from four eukaryotic interactomes. *PLoS Comput. Biol.* 2:e100.
4. Tompa, P. 2005. The interplay between structure and function in intrinsically unstructured proteins. *FEBS Lett.* 579:3346–3354.
5. Wright, P. E., and H. J. Dyson. 2009. Linking folding and binding. *Curr. Opin. Struct. Biol.* 19:31–38.
6. Uversky, V. N. 2013. A decade and a half of protein intrinsic disorder: biology still waits for physics. *Protein Sci.* 22:693–724.
7. Chen, J. 2012. Towards the physical basis of how intrinsic disorder mediates protein function. *Arch. Biochem. Biophys.* 524:123–131.
8. Mittag, T., L. E. Kay, and J. D. Forman-Kay. 2010. Protein dynamics and conformational disorder in molecular recognition. *J. Mol. Recognit.* 23:105–116.
9. Liu, Z., and Y. Huang. 2014. Advantages of proteins being disordered. *Protein Sci.* 23:539–550.
10. Das, R. K., K. M. Ruff, and R. V. Pappu. 2015. Relating sequence encoded information to form and function of intrinsically disordered proteins. *Curr. Opin. Struct. Biol.* 32:102–112.
11. Shoemaker, B. A., J. J. Portman, and P. G. Wolynes. 2000. Speeding molecular recognition by using the folding funnel: the fly-casting mechanism. *Proc. Natl. Acad. Sci. USA.* 97:8868–8873.
12. Huang, Y., and Z. Liu. 2009. Kinetic advantage of intrinsically disordered proteins in coupled folding-binding process: a critical assessment of the "fly-casting" mechanism. *J. Mol. Biol.* 393:1143–1159.
13. Dyson, H. J., and P. E. Wright. 2005. Intrinsically unstructured proteins and their functions. *Nat. Rev. Mol. Cell Biol.* 6:197–208.

14. Iakoucheva, L. M., P. Radivojac, …, A. K. Dunker. 2004. The importance of intrinsic disorder for protein phosphorylation. *Nucleic Acids Res.* 32:1037–1049.

15. Bah, A., and J. D. Forman-Kay. 2016. Modulation of intrinsically disordered protein function by post-translational modifications. *J. Biol. Chem.* 291:6696–6705.

16. Romero, P. R., S. Zaidi, …, A. K. Dunker. 2006. Alternative splicing in concert with protein intrinsic disorder enables increased functional diversity in multicellular organisms. *Proc. Natl. Acad. Sci. USA.* 103:8390–8395.

17. Babu, M. M. 2016. The contribution of intrinsically disordered regions to protein function, cellular complexity, and human disease. *Biochem. Soc. Trans.* 44:1185–1200.

18. Tompa, P., C. Szász, and L. Buday. 2005. Structural disorder throws new light on moonlighting. *Trends Biochem. Sci.* 30:484–489.

19. Fuxreiter, M., P. Tompa, …, F. J. Asturias. 2008. Malleable machines take shape in eukaryotic transcriptional regulation. *Nat. Chem. Biol.* 4:728–737.

20. Ward, J. J., J. S. Sodhi, …, D. T. Jones. 2004. Prediction and functional analysis of native disorder in proteins from the three kingdoms of life. *J. Mol. Biol.* 337:635–645.

21. Pancsa, R., and P. Tompa. 2012. Structural disorder in eukaryotes. *PLoS One.* 7:e34687.

22. Liu, J., N. B. Perumal, …, A. K. Dunker. 2006. Intrinsic disorder in transcription factors. *Biochemistry.* 45:6873–6888.

23. van der Lee, R., M. Buljan, …, M. M. Babu. 2014. Classification of intrinsically disordered regions and proteins. *Chem. Rev.* 114:6589–6631.

24. Milles, S., D. Mercadante, …, E. A. Lemke. 2015. Plasticity of an ultrafast interaction between nucleoporins and nuclear transport receptors. *Cell.* 163:734–745.

25. Fisher, C. K., and C. M. Stultz. 2011. Constructing ensembles for intrinsically disordered proteins. *Curr. Opin. Struct. Biol.* 21:426–431.

26. Shammas, S. L., M. D. Crabtree, …, J. Clarke. 2016. Insights into coupled folding and binding mechanisms from kinetic studies. *J. Biol. Chem.* 291:6689–6695.

27. Geierhaas, C. D., X. Salvatella, …, M. Vendruscolo. 2008. Characterisation of transition state structures for protein folding using 'high', 'medium' and 'low' Φ-values. *Protein Eng. Des. Sel.* 21:215–222.

28. Fersht, A. 1999. Structure and Mechanicsm in Protein Science - A Guide to Enzyme Catalysis and Protein Folding. W. H. Freeman, New York.

29. Fersht, A. R., A. Matouschek, and L. Serrano. 1992. The folding of an enzyme. I. Theory of protein engineering analysis of stability and pathway of protein folding. *J. Mol. Biol.* 224:771–782.

30. Bachmann, A., D. Wildemann, …, T. Kiefhaber. 2011. Mapping backbone and side-chain interactions in the transition state of a coupled protein folding and binding reaction. *Proc. Natl. Acad. Sci. USA.* 108:3952–3957.

31. Rogers, J. M., V. Oleinikovas, …, J. Clarke. 2014. Interplay between partner and ligand facilitates the folding and binding of an intrinsically disordered protein. *Proc. Natl. Acad. Sci. USA.* 111:15420–15425.

32. Giri, R., A. Morrone, …, S. Gianni. 2013. Structure of the transition state for the binding of c-Myb and KIX highlights an unexpected order for a disordered system. *Proc. Natl. Acad. Sci. USA.* 110:14942–14947.

33. Hill, S. A., L. G. Kwa, …, J. Clarke. 2014. Mechanism of assembly of the non-covalent spectrin tetramerization domain from intrinsically disordered partners. *J. Mol. Biol.* 426:21–35.

34. Dogan, J., X. Mu, …, P. Jemth. 2013. The transition state structure for coupled binding and folding of disordered protein domains. *Sci. Rep.* 3:2076.

35. Toto, A., and S. Gianni. 2016. Mutational analysis of the binding-induced folding reaction of the Mixed-Lineage Leukemia protein to the KIX domain. *Biochemistry.* 55:3957–3962.

36. Kee, B. L., J. Arias, and M. R. Montminy. 1996. Adaptor-mediated recruitment of RNA polymerase II to a signal-dependent activator. *J. Biol. Chem.* 271:2373–2375.

37. Mayr, B., and M. Montminy. 2001. Transcriptional regulation by the phosphorylation-dependent factor CREB. *Nat. Rev. Mol. Cell Biol.* 2:599–609.

38. Nakajima, T., C. Uchida, …, M. Montminy. 1997. Analysis of a cAMP-responsive activator reveals a two-component mechanism for transcriptional induction via signal-dependent factors. *Genes Dev.* 11:738–747.

39. Thakur, J. K., A. Yadav, and G. Yadav. 2014. Molecular recognition by the KIX domain and its role in gene regulation. *Nucleic Acids Res.* 42:2112–2125.

40. Radhakrishnan, I., G. C. Pérez-Alvarado, …, P. E. Wright. 1997. Solution structure of the KIX domain of CBP bound to the transactivation domain of CREB: a model for activator:coactivator interactions. *Cell.* 91:741–752.

41. Parker, D., U. S. Jhala, …, M. Montminy. 1998. Analysis of an activator:coactivator complex reveals an essential role for secondary structure in transcriptional activation. *Mol. Cell.* 2:353–359.

42. Dahal, L., S. L. Shammas, and J. Clarke. 2017. Phosphorylation of the IDP KID modulates affinity for KIX by increasing the lifetime of the complex. *Biophys. J.* 113:2706–2712.

43. Sugase, K., H. J. Dyson, and P. E. Wright. 2007. Mechanism of coupled folding and binding of an intrinsically disordered protein. *Nature.* 447:1021–1025.

44. Shammas, S. L., A. J. Travis, and J. Clarke. 2014. Allostery within a transcription coactivator is predominantly mediated through dissociation rate constants. *Proc. Natl. Acad. Sci. USA.* 111:12055–12060.

45. Huang, Y., and Z. Liu. 2010. Nonnative interactions in coupled folding and binding processes of intrinsically disordered proteins. *PLoS One.* 5:e15375.

46. Huang, Y., and Z. Liu. 2010. Smoothing molecular interactions: the "kinetic buffer" effect of intrinsically disordered proteins. *Proteins.* 78:3251–3259.

47. Turjanski, A. G., J. S. Gutkind, …, G. Hummer. 2008. Binding-induced folding of a natively unstructured transcription factor. *PLOS Comput. Biol.* 4:e1000060.

48. Ganguly, D., and J. Chen. 2009. Atomistic details of the disordered states of KID and pKID. Implications in coupled binding and folding. *J. Am. Chem. Soc.* 131:5214–5223.

49. Ganguly, D., and J. Chen. 2011. Topology-based modeling of intrinsically disordered proteins: balancing intrinsic folding and intermolecular interactions. *Proteins.* 79:1251–1266.

50. Kurcinski, M., A. Kolinski, and S. Kmiecik. 2014. Mechanism of folding and binding of an intrinsically disordered protein as revealed by ab initio simulations. *J. Chem. Theory Comput.* 10:2224–2231.

51. Shammas, S. L., A. J. Travis, and J. Clarke. 2013. Remarkably fast coupled folding and binding of the intrinsically disordered transactivation domain of cMyb to CBP KIX. *J. Phys. Chem. B.* 117:13346–13356.

52. Fersht, A. R. 1995. Characterizing transition states in protein folding: an essential step in the puzzle. *Curr. Opin. Struct. Biol.* 5:79–84.

53. Fersht, A. R., and S. Sato. 2004. Φ-value analysis and the nature of protein-folding transition states. *Proc. Natl. Acad. Sci. USA.* 101:7976–7981.

54. Scott, K. A., D. O. V. Alonso, …, V. Daggett. 2007. Conformational entropy of alanine versus glycine in protein denatured states. *Proc. Natl. Acad. Sci. USA.* 104:2661–2666.

55. Zor, T., B. M. Mayr, …, P. E. Wright. 2002. Roles of phosphorylation and helix propensity in the binding of the KIX domain of CREB-binding protein by constitutive (c-Myb) and inducible (CREB) activators. *J. Biol. Chem.* 277:42241–42248.

56. Solt, I., C. Magyar, …, M. Fuxreiter. 2006. Phosphorylation-induced transient intrinsic structure in the kinase-inducible domain of CREB facilitates its recognition by the KIX domain of CBP. *Proteins.* 64:749–757.

57. Bomblies, R., M. P. Luitz, and M. Zacharias. 2016. Mechanism of pKID/KIX association studied by molecular dynamics free energy simulations. *J. Phys. Chem. B.* 120:8186–8192.

58. Radhakrishnan, I., G. C. Pérez-Alvarado, …, P. E. Wright. 1998. Conformational preferences in the Ser133-phosphorylated and non-phosphorylated forms of the kinase inducible transactivation domain of CREB. *FEBS Lett.* 430:317–322.

59. Umezawa, K., J. Ikebe, …, J. Higo. 2012. Conformational ensembles of an intrinsically disordered protein pKID with and without a KIX domain in explicit solvent investigated by all-atom multicanonical molecular dynamics. *Biomolecules.* 2:104–121.

60. Chen, H.-F. 2009. Molecular dynamics simulation of phosphorylated KID post-translational modification. *PLoS One.* 4:e6516.

61. Umezawa, K., J. Ohnuki, …, M. Takano. 2016. Intrinsic disorder accelerates dissociation rather than association. *Proteins.* 84:1124–1133.

62. De Sancho, D., and R. B. Best. 2012. Modulation of an IDP binding mechanism and rates by helix propensity and non-native interactions: association of HIF1α with CBP. *Mol. Biosyst.* 8:256–267.

63. Tompa, P., and M. Fuxreiter. 2008. Fuzzy complexes: polymorphism and structural disorder in protein-protein interactions. *Trends Biochem. Sci.* 33:2–8.

64. Schneider, R., D. Maurin, …, M. Blackledge. 2015. Visualizing the molecular recognition trajectory of an intrinsically disordered protein using multinuclear relaxation dispersion NMR. *J. Am. Chem. Soc.* 137:1220–1229.

65. Hammes, G. G., Y. C. Chang, and T. G. Oas. 2009. Conformational selection or induced fit: a flux description of reaction mechanism. *Proc. Natl. Acad. Sci. USA.* 106:13737–13741.

66. Daniels, K. G., N. K. Tonthat, …, T. G. Oas. 2014. Ligand concentration regulates the pathways of coupled protein folding and binding. *J. Am. Chem. Soc.* 136:822–825.

67. Gianni, S., A. Morrone, …, M. Brunori. 2012. A folding-after-binding mechanism describes the recognition between the transactivation domain of c-Myb and the KIX domain of the CREB-binding protein. *Biochem. Biophys. Res. Commun.* 428:205–209.

68. Haq, S. R., C. N. Chi, …, P. Jemth. 2012. Side-chain interactions form late and cooperatively in the binding reaction between disordered peptides and PDZ domains. *J. Am. Chem. Soc.* 134:599–605.

69. Odoux, A., D. Jindal, …, W. Xu. 2016. Experimental and molecular dynamics studies showed that CBP KIX mutation affects the stability of CBP:c-Myb complex. *Comput. Biol. Chem.* 62:47–59.

70. Tetley, G. J. N., H. R. Mott, …, D. Owen. 2017. A dock and coalesce mechanism driven by hydrophobic interactions governs Cdc42 binding with its effector protein ACK. *J. Biol.Chem.* 292:11361–11373.

71. Chu, W. T., J. Clarke, …, J. Wang. 2017. Role of non-native electrostatic interactions in the coupled folding and binding of PUMA with Mcl-1. *PLOS Comput. Biol.* 13:e1005468.

72. Mollica, L., L. M. Bessa, …, R. Schneider. 2016. Binding mechanisms of intrinsically disordered proteins: theory, simulation, and experiment. *Front. Mol. Biosci.* 3:52.

73. Wang, Y., X. Chu, …, J. Wang. 2013. Multiscaled exploration of coupled folding and binding of an intrinsically disordered molecular recognition element in measles virus nucleoprotein. *Proc. Natl. Acad. Sci. USA.* 110:E3743–E3752.

74. Jensen, M. R., K. Houben, …, M. Blackledge. 2008. Quantitative conformational analysis of partially folded proteins from residual dipolar couplings: application to the molecular recognition element of Sendai virus nucleoprotein. *J. Am. Chem. Soc.* 130:8055–8061.

75. Arai, M., K. Sugase, …, P. E. Wright. 2015. Conformational propensities of intrinsically disordered proteins influence the mechanism of binding and folding. *Proc. Natl. Acad. Sci. USA.* 112:9614–9619.

76. Ithuralde, R. E., A. E. Roitberg, and A. G. Turjanski. 2016. Structured and unstructured binding of an intrinsically disordered protein as revealed by atomistic simulations. *J. Am. Chem. Soc.* 138:8742–8751.

77. Dogan, J., and P. Jemth. 2014. Only kinetics can prove conformational selection. *Biophys. J.* 107:1997–1998.

78. Gibbs, E. B., and S. A. Showalter. 2015. Quantitative biophysical characterization of intrinsically disordered proteins. *Biochemistry.* 54:1314–1326.

79. Dosnon, M., D. Bonetti, …, S. Gianni. 2015. Demonstration of a folding after binding mechanism in the recognition between the measles virus NTAIL and X domains. *ACS Chem. Biol.* 10:795–802.

80. Toto, A., C. Camilloni, …, S. Gianni. 2016. Molecular recognition by templated folding of an intrinsically disordered protein. *Sci. Rep.* 6:21994.

81. Ganguly, D., W. Zhang, and J. Chen. 2013. Electrostatically accelerated encounter and folding for facile recognition of intrinsically disordered proteins. *PLOS Comput. Biol.* 9:e1003363.

82. Iešmantavičius, V., J. Dogan, …, M. Kjaergaard. 2014. Helical propensity in an intrinsically disordered protein accelerates ligand binding. *Angew. Chem. Int. Ed. Engl.* 53:1548–1551.

Biophysical Journal, Volume *113*

Supplemental Information

pKID Binds to KIX via an Unstructured Transition State with Nonnative Interactions

Liza Dahal, Tristan O.C. Kwan, Sarah L. Shammas, and Jane Clarke

Supplementary Tables

Table S1. Kinetic and thermodynamic rate constants for the binding of wild-type and interface mutants of pKID to KIX. The errors for $k_{ass,fast}$, k_{diss} and K_d represent errors from the fit. Φ-values were calculated using both kinetic and equilibrium measurements and the errors propagated using standard equations.

pKID	$k_{ass,fast}$ (μM^{-1}s^{-1})	k_{diss} (s^{-1})	K_{dEqub} (μM)	K_{dKin} (μM)	$\Delta\Delta G_{Equb}$ (kcal.mol^{-1})	$\Delta\Delta G_{Kin}$ (kcal.mol^{-1})	Φ_{Equb}	Φ_{Kin}
**Interface mutants**								
Wild-type	7.3±0.3	0.81±0.01	0.11±0.02	0.11±0.02	-	-	-	-
S121A	6.9±1.2	0.71±0.01	0.12±0.02	0.10±0.02	0.05±0.14	-0.04±0.10	0.65±2.75*	-0.75±4.14*
R124A	8.4±1.6	0.51±0.1	0.06±0.01	0.06±0.02	-0.34±0.14	-0.34±0.16	0.23±0.34	0.23±0.26
R125A	6.3±0.5	2.17±0.04	0.22±0.02	0.34±0.03	0.39±0.11	0.64±0.05	0.21±0.14	0.13±0.07
L128A	7.9±0.3	15.7±1.3	2.49±0.06	2.00±0.16	1.75±0.10	1.62±0.05	-0.03±0.02	-0.03±0.02
R131A	9.5±1	7.66±1.04	1.23±0.12	0.81±0.14	1.36±0.12	1.12±0.10	-0.11±0.05	-0.13±0.07
ΔpS133	7.6±0.8	56.7±7.5	28.7±1.2	7.5±1.3	3.13±0.10	2.37±0.10	-0.01±0.02	-0.01±0.03
Y134A	†	†	110.6±9.8	†	3.89±0.11	†	†	†
I137A	4.5±0.5	43.9±7.2	21.6±0.50	9.73±1.92	2.97±0.10	2.52±0.11	0.09±0.02	0.11±0.02
L138A	2.8±1.1	13.2±1.4	6.30±0.30	4.73±1.92	2.28±0.11	2.11±0.23	0.24±0.10	0.26±0.08
D140A	5.3±0.7	2.06±0.09	0.26±0.04	0.39±0.05	0.48±0.13	0.70±0.08	0.37±0.19	0.26±0.08
S142A	11.3±1	0.83±0.03	0.12±0.01	0.07±0.01	0.05±0.11	-0.23±0.06	-5.20±11.6*	1.06±0.10*

*Mutants where Φ-values are unreliable and not reported in the main text as they show $\Delta\Delta G_{Equb}$ and/or $\Delta\Delta G_{Kin}$ < 0.34 kcal mol^{-1}. (See main paper for discussion.)
† Kinetic measurements could not be obtained, as good signal change upon binding was not observed.

Table S2. Kinetic and thermodynamic rate constants for the binding of solvent exposed mutants of pKID to KIX. The errors for $k_{ass,fast}$, k_{diss} and K_d represent errors from the fit. Φ-values were calculated using both kinetic and equilibrium measurements and the errors propagated using standard equations. $\Delta\Delta G$ values for Ala-Gly composite mutations are shown.

pKID	$k_{ass,fast}$ ($\mu M^{-1}s^{-1}$)	k_{diss} (s^{-1})	K_{dEqub} (μM)	K_{dKin} (μM)	$\Delta\Delta G_{Equb}$ ($kcal.mol^{-1}$)	$\Delta\Delta G_{Kin}$ ($kcal.mol^{-1}$)	Φ_{Equb}	Φ_{Kin}
**Solvent Exposed mutants**								
T119A	7.3±0.7	0.88±0.01	0.15±0.02	0.12±0.01	0.17±0.13	0.05±0.06	-	-
A119G	7.1±1.1	0.67±0.02	0.08±0.02	0.09±0.01	-0.35±0.16	-0.14±0.10	-0.04±0.29*	0.11±0.83*
E126A	10.5±1.7	0.97±0.01	0.17±0.01	0.09±0.01	0.24±0.11	-0.10±0.09	-	-
A126G	7.6±0.9	2.09±0.02	0.25±0.02	0.28±0.03	0.22±0.06	0.61±0.11	0.84±0.56*	0.30±0.13*
P132A	7.7±0.4	0.99±0.05	0.07±0.02	0.13±0.01	-0.25±0.19	0.08±0.05	-	-
A132G	8.3±0.2	3.22±0.03	0.44±0.03	0.39±0.01	1.03±0.17	0.62±0.04	-0.04±0.03	-0.07±0.06
R135A	10.5±0.6	2.50±0.20	0.10±0.02	0.24±0.02	-0.05±0.15	0.43±0.06	-	-
A135G	8±0.4	10.1±0.30	1.16±0.05	1.26±0.07	1.38±0.12	0.94±0.06	0.11±0.03	0.16±0.04
K136A	10.2±0.9	0.94±0.03	0.10±0.02	0.09±0.01	-0.05±0.15	-0.10±0.06	-	-
A136G	8.3±1.5	10.5±0.80	2.16±0.11	1.27±0.25	1.73±0.12	1.47±0.12	0.07±0.07	0.08±0.07
S143A	9.7±1	0.71±0.01	0.07±0.01	0.07±0.01	-0.25±0.13	-0.23±0.06	-	-
A143G	6.3±0.6	1.20±0.10	0.09±0.01	0.19±0.02	0.14±0.10	0.54±0.09	1.72±1.36*	0.45±0.09*

*Mutants where Φ-values are unreliable and not reported in the main text as they show $\Delta\Delta G_{Equb}$ and/or $\Delta\Delta G_{Kin} < 0.34$ kcal mol^{-1}. (See main paper for discussion.)

Supplementary Figures

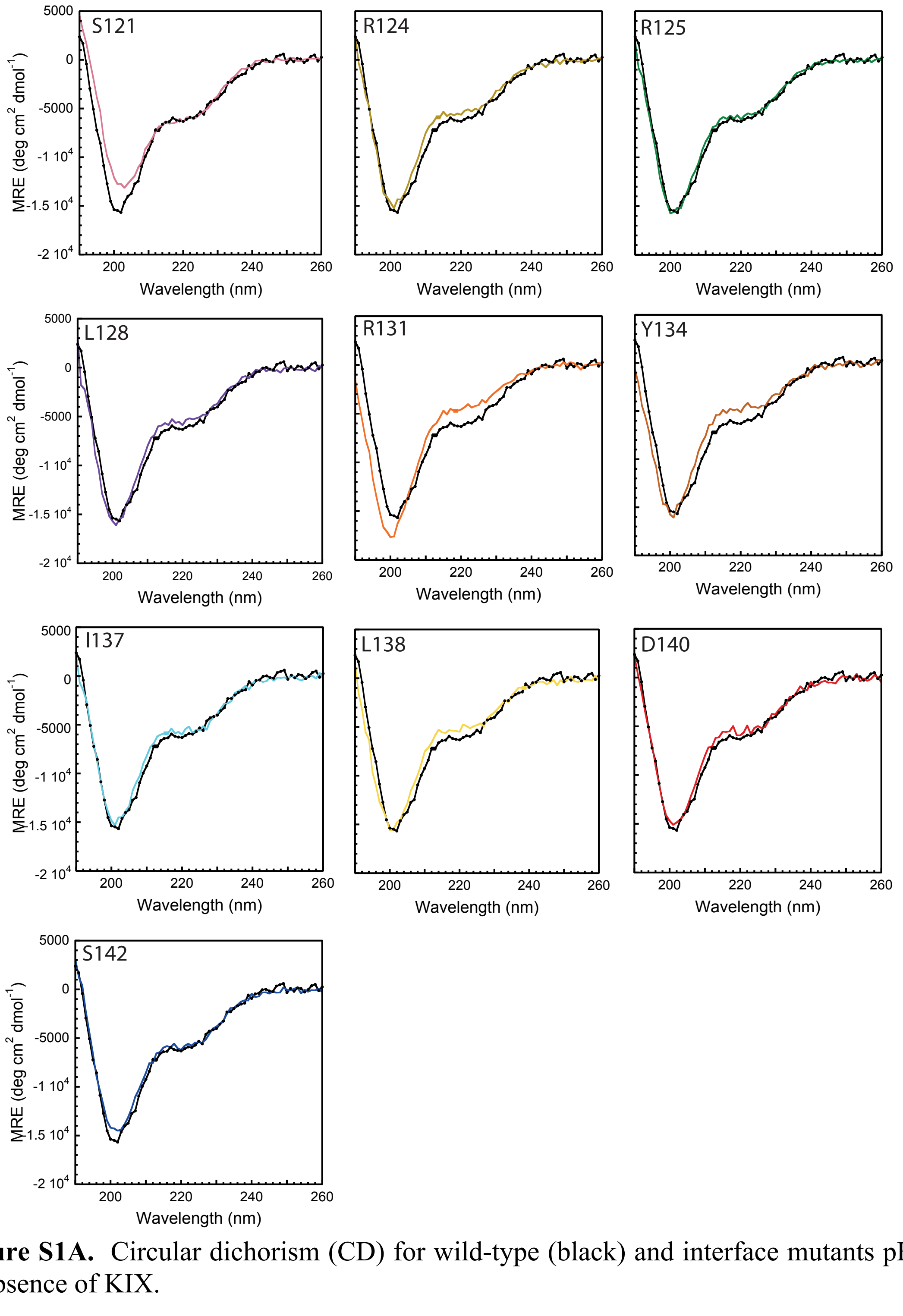

Figure S1A. Circular dichorism (CD) for wild-type (black) and interface mutants pKID in absence of KIX.

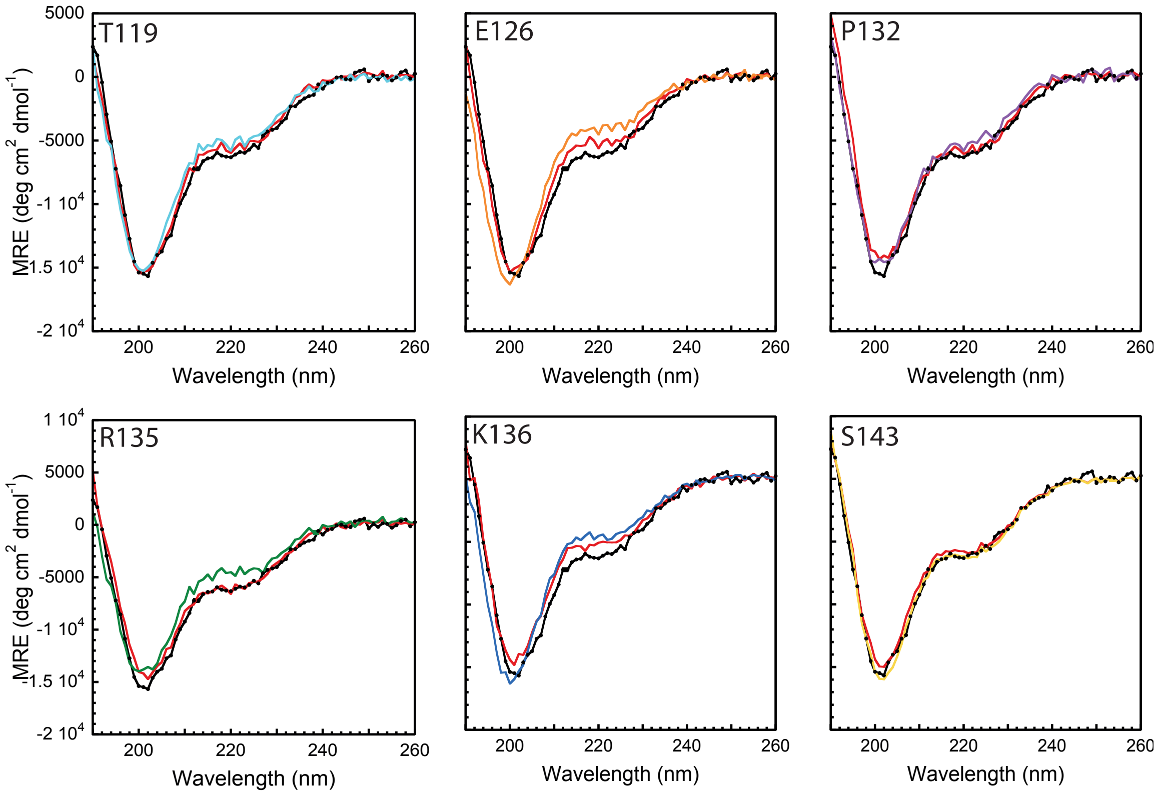

Figure S1B. Circular dichorism (CD) for wild-type (black) and solvent exposed mutants (Ala and Gly) of pKID in absence of KIX. Ala mutations are shown in red and Gly mutants are shown as labeled.

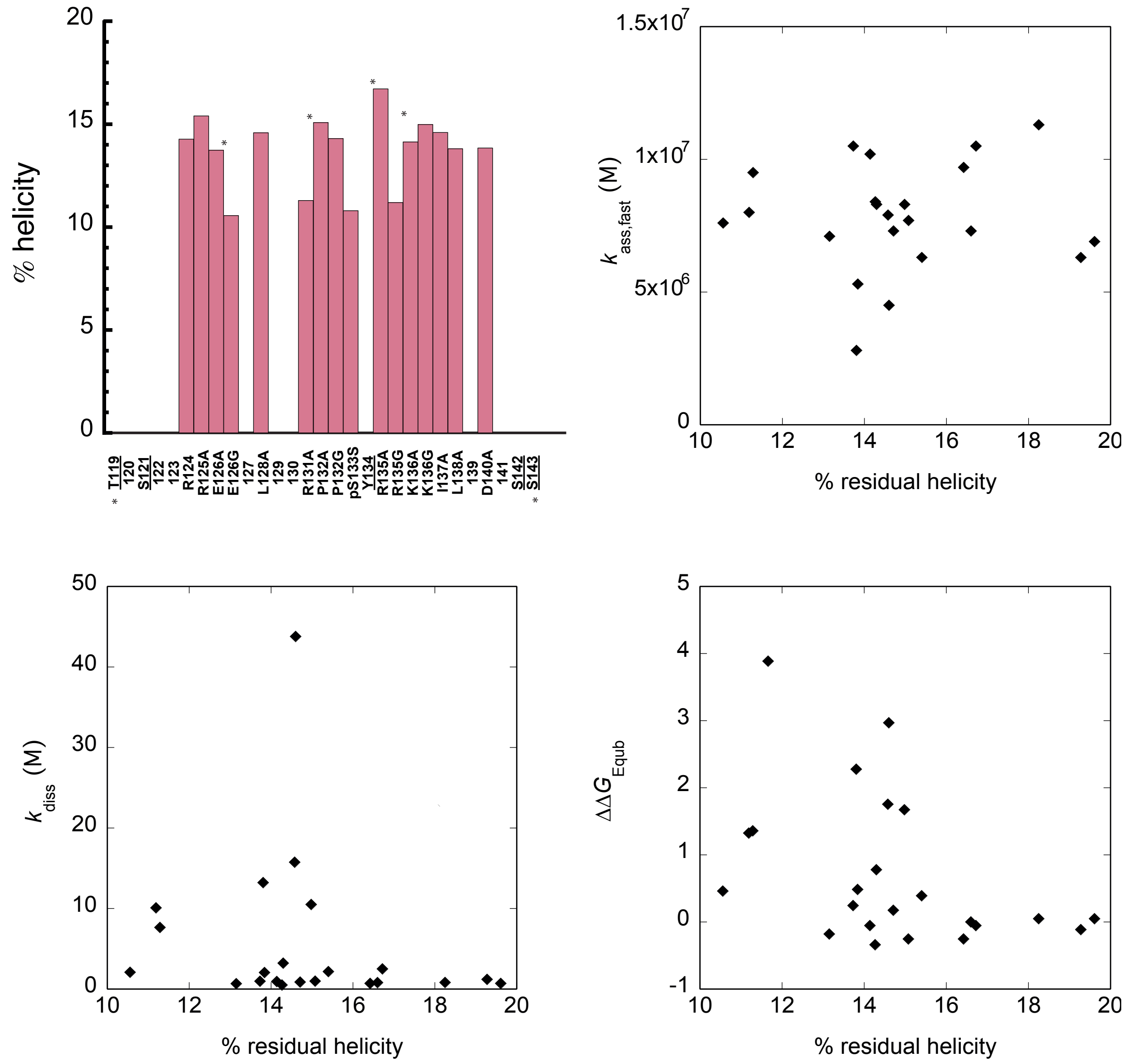

Figure S2. Percentage (%) residual helicity of pKID mutants. (Top left panel) % helicity of interface and solvent exposed pKID mutants used to calculate the Φ values are shown as bar charts. The * on top of the bar highlights the solvent exposed (Ala-Gly) mutants in the bar chart. There is no detectable relationship between residual helicity and any biophysical parameters: (Top right panel) fast association kinetic rate ($k_{ass,fast}$) plotted against % helicity for all pKID mutants. (Bottom left panel) dissociation kinetic rate (k_{diss}) plotted against % helicity for all pKID mutants. (Bottom right panel) $\Delta\Delta G_{Equb}$ plotted against % helicity for all pKID mutants.

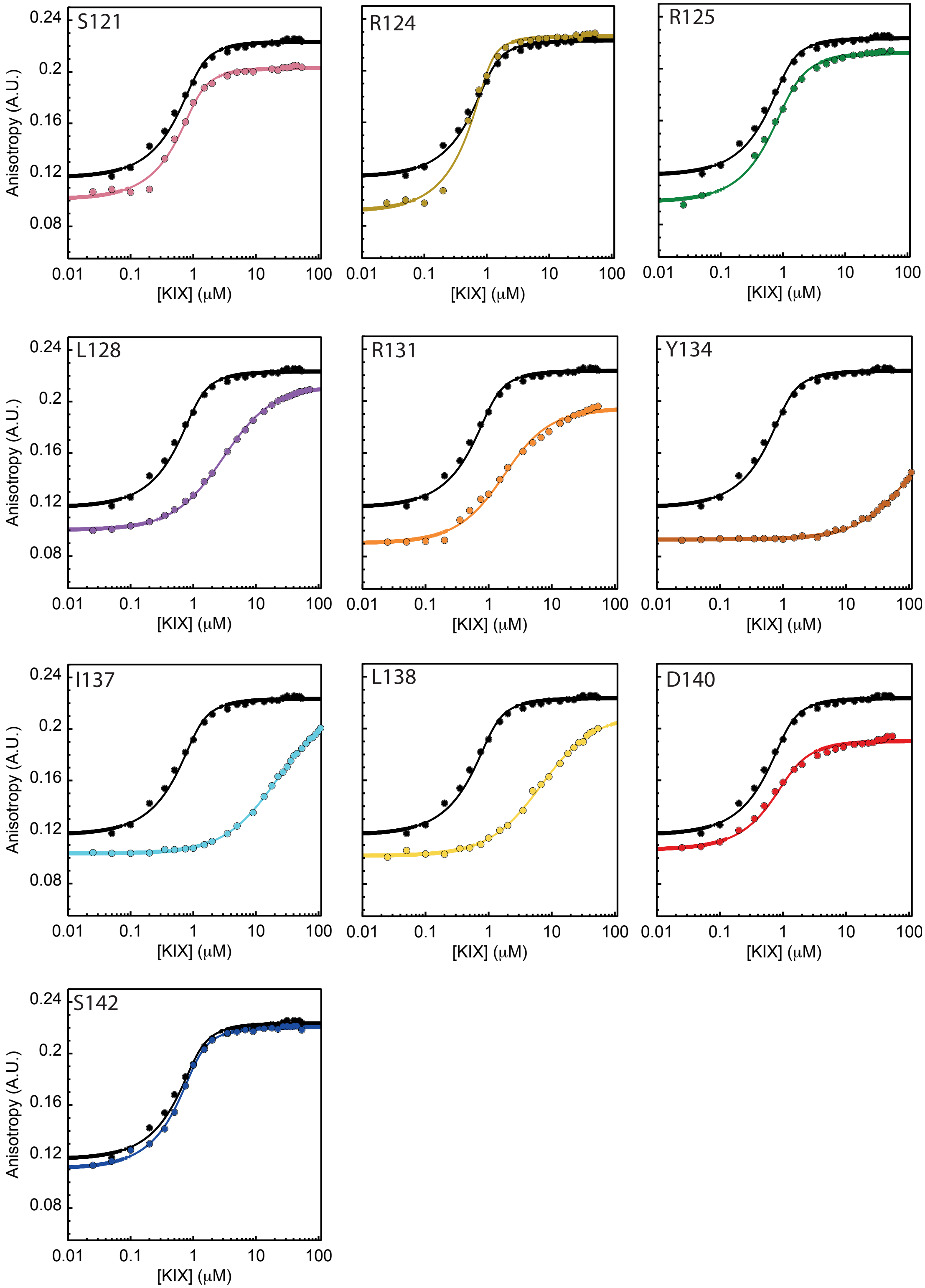

Figure S3A. Equilibrium anisotropy binding curves for the interface mutants of pKID. Wild-type is shown in black.

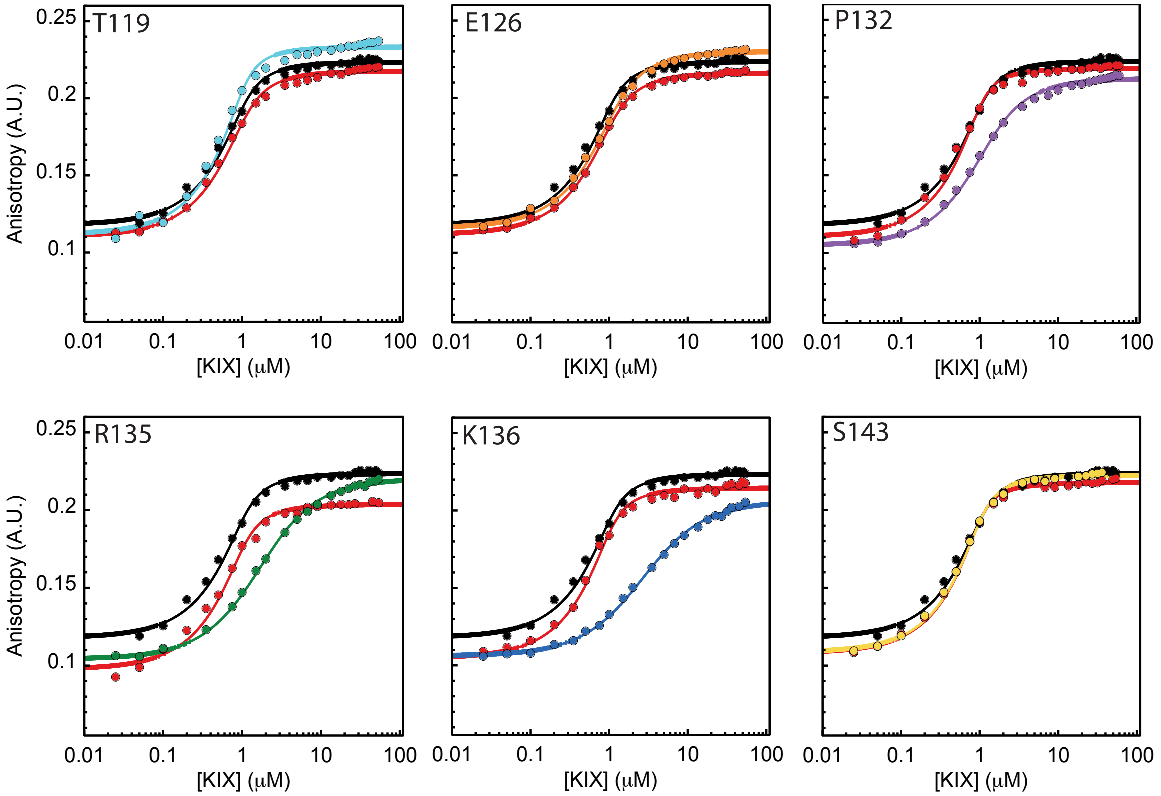

Figure S3B. Equilibrium anisotropy binding curves for the solvent exposed mutants of pKID. Wild-type is shown in black and all Ala mutants are shown in red. Gly mutants are as labeled.

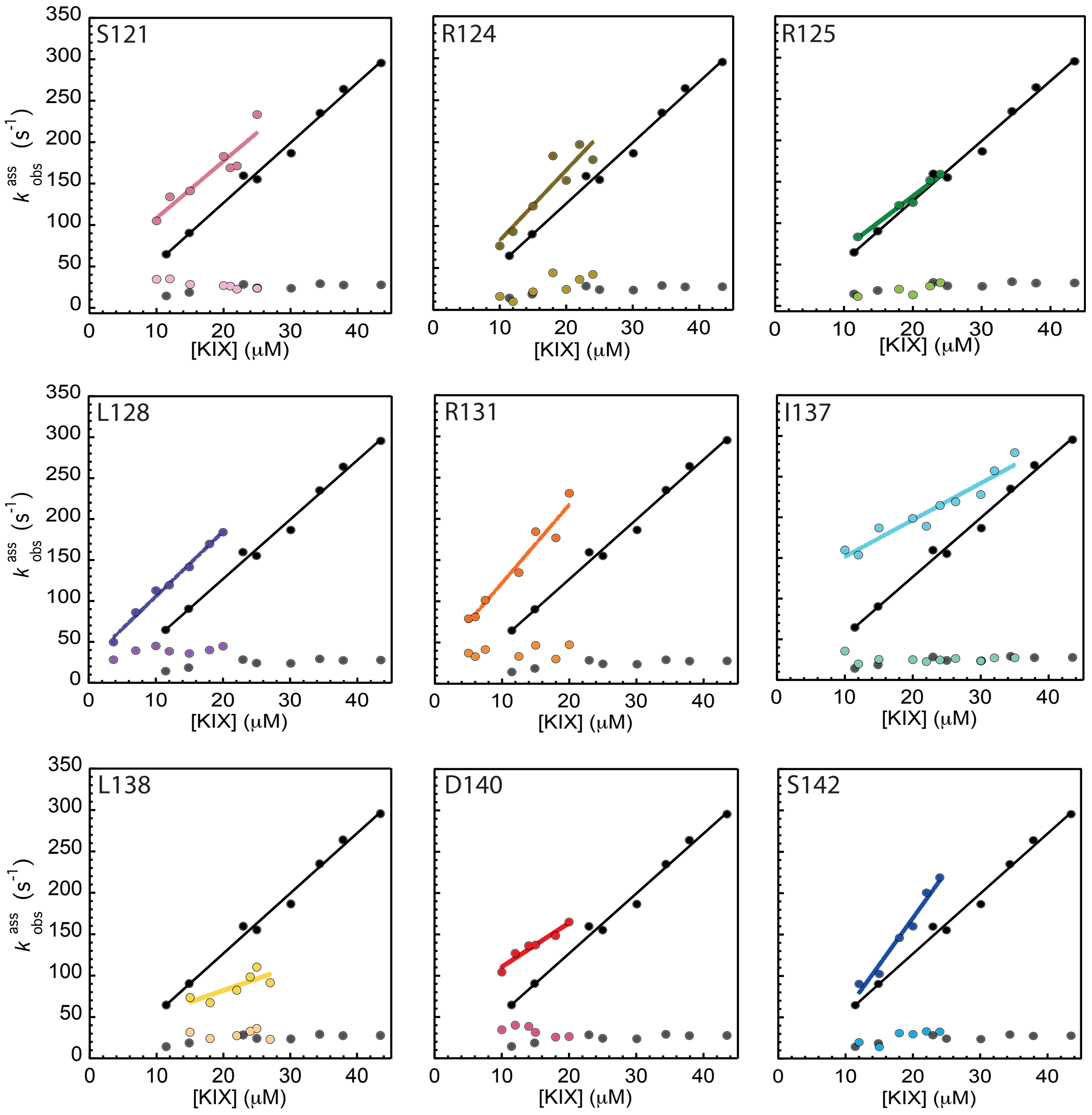

Figure S4A. Observed association kinetics rate of pKID and KIX for the interface mutations under pseudo-first order conditions with KIX in excess. Wild-type data is shown in black.

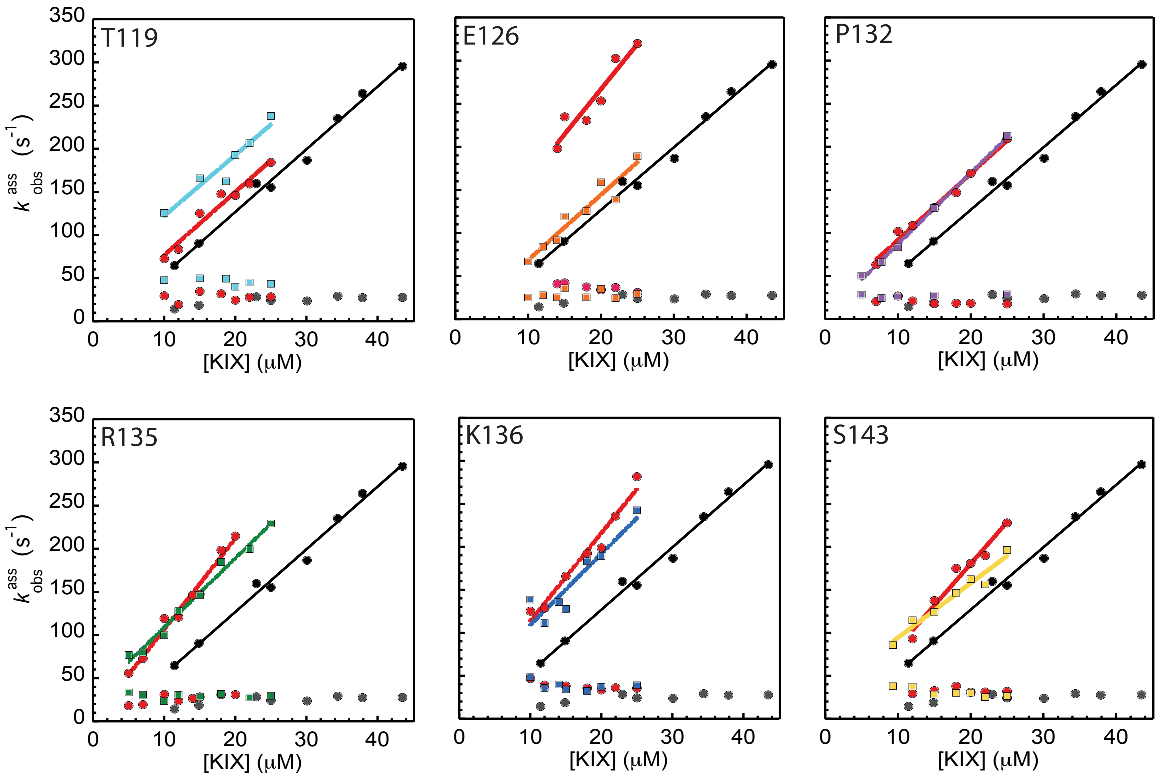

Figure S4B. Observed association kinetics rate of pKID and KIX for the solvent exposed pKID mutants obtained under pseudo-first order conditions with KIX in excess. Wild-type is shown in black and all Ala mutants are shown in red. Gly mutants are shown as labeled.

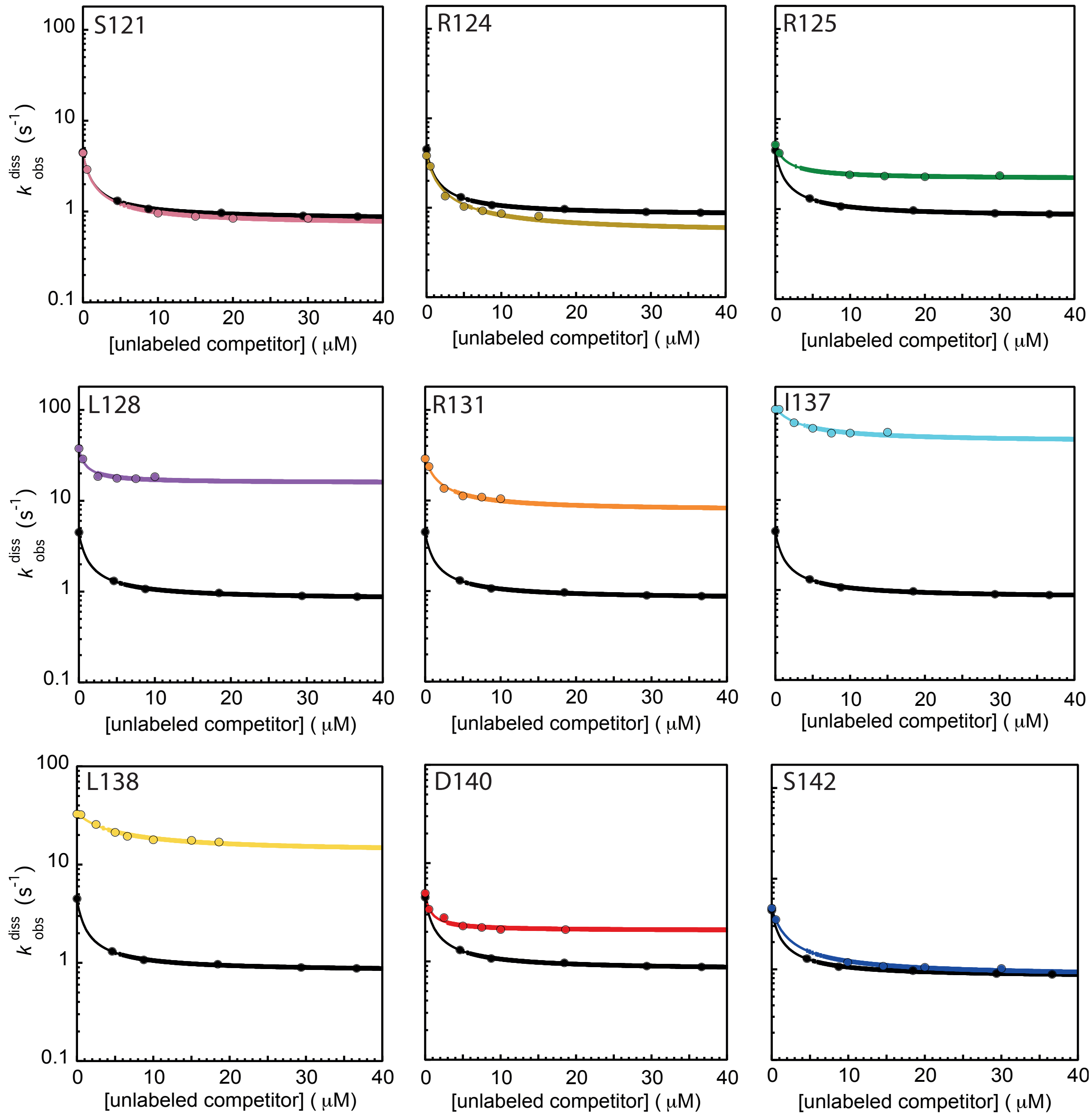

Figure S5A. Observed dissociation kinetics rate of wild-type (black) and interface pKID mutants from KIX obtained using cMybTAD as a competitor..

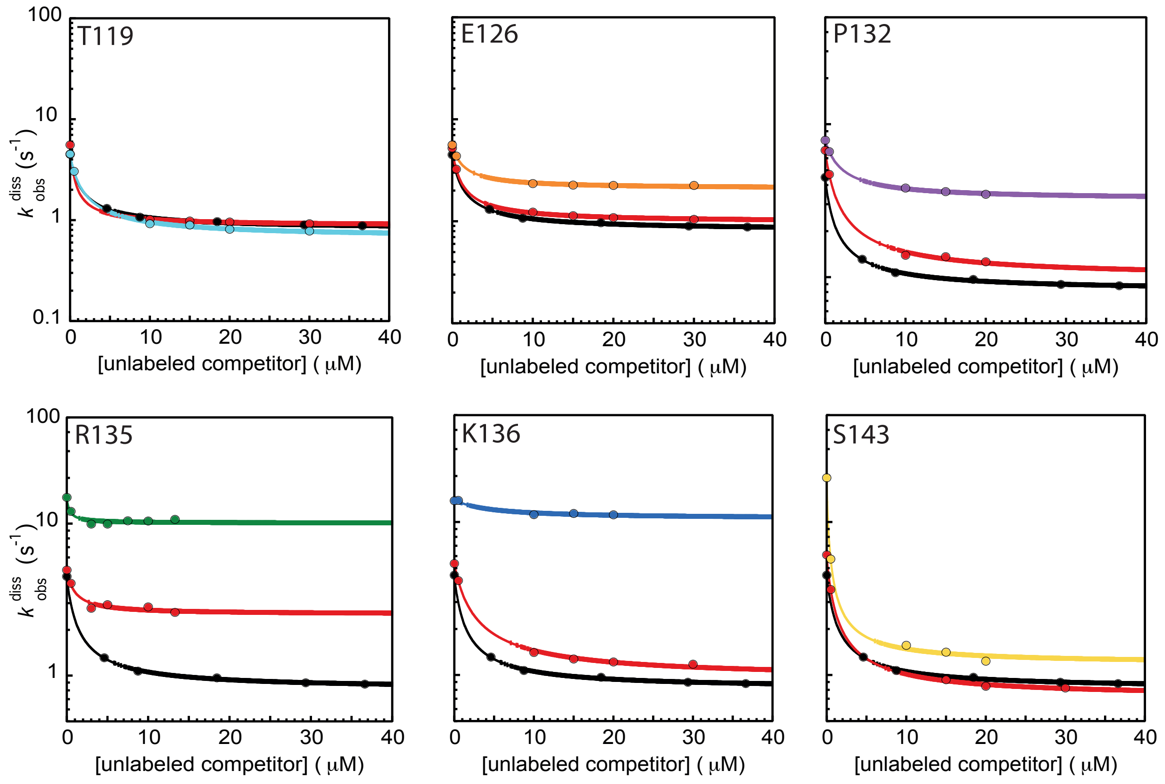

Figure S5B. Observed dissociation kinetics rate of wild-type (black spheres) and solvent exposed pKID mutants from KIX obtained using cMybTAD as a competitor. Wild-type is shown as black and all Ala mutants are shown in red. Gly mutants are shown as labeled.

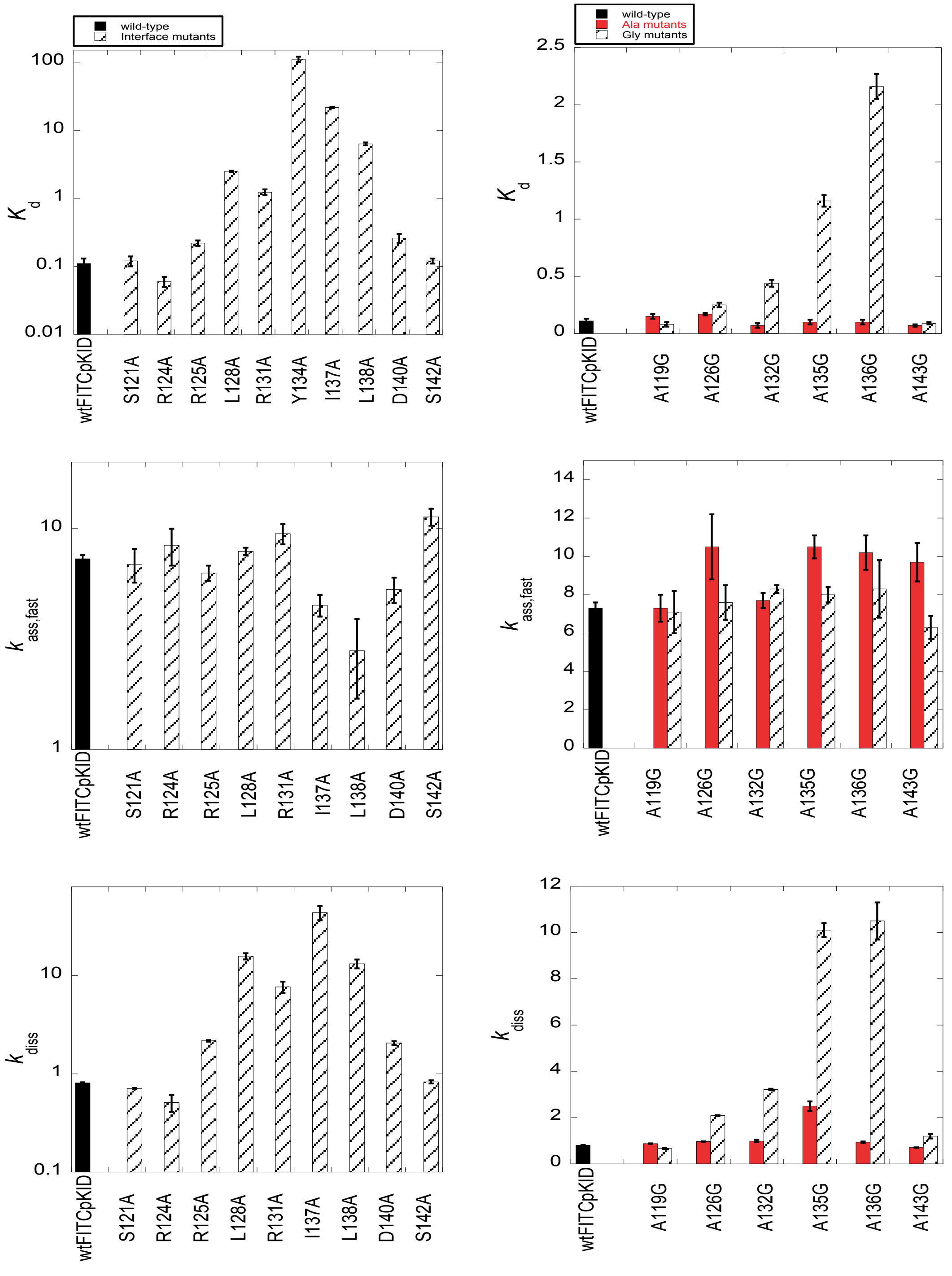

Figure S6. Bar chart showing equilibrium binding (K_d), fast association kinetics ($k_{ass,fast}$) and dissociation kinetics (k_{diss}) comparison of all mutants of pKID. Wild type is shown in filled black bar for all graphs. The bar charts on the left side show all interface mutants (hatched bar). The bar charts on the right show all Ala-Gly mutants. The Ala mutants are shown in red and the Gly mutants in hatched.

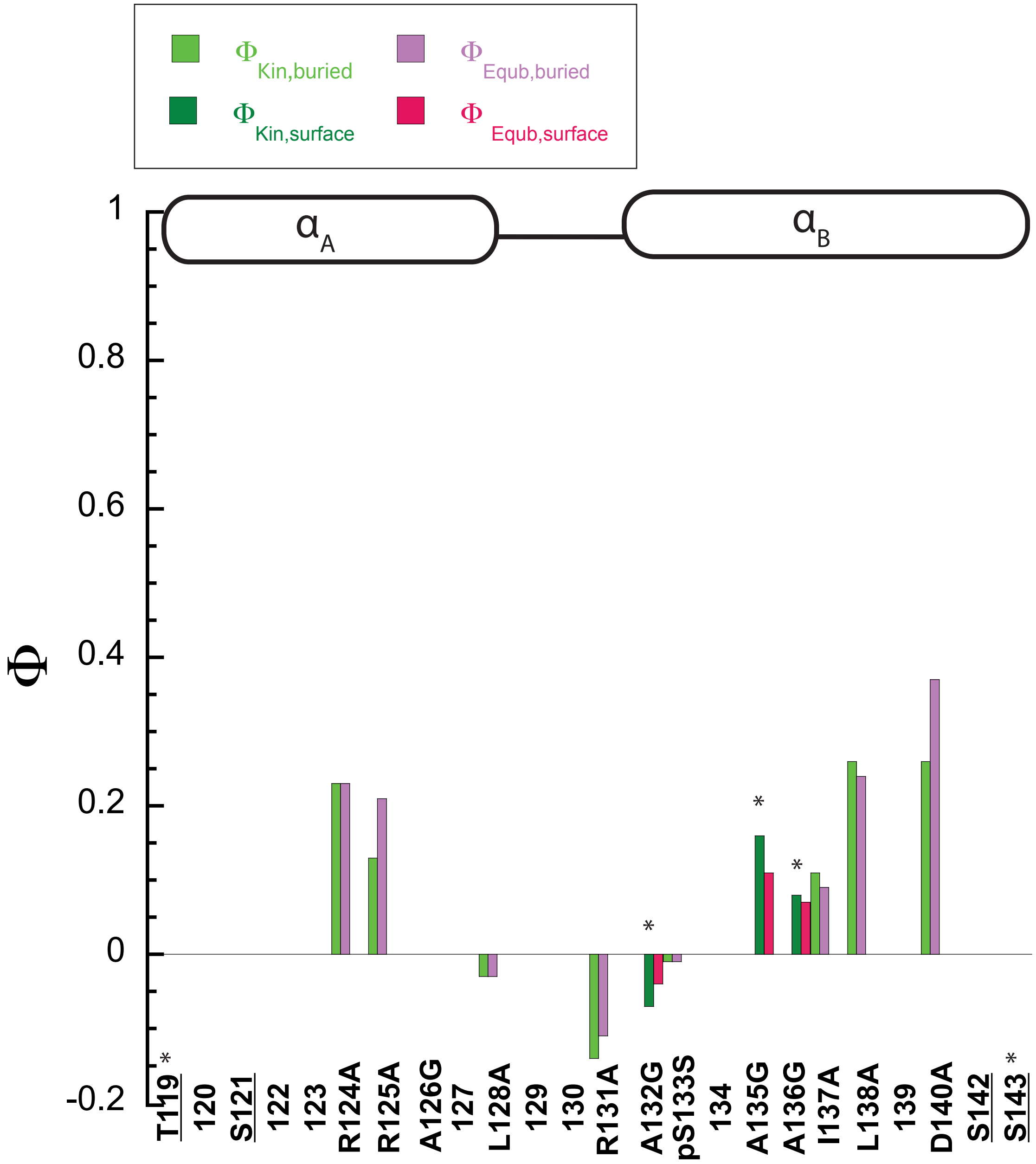

Figure S7. The pattern of Φ-values is the same whether using equilibrium or kinetic $\Delta\Delta G$. Bar chart showing comparison between Φ-values calculated using kinetic and equilibrium measurements. The Φ-values calculated using kinetic for interface mutants is shown in pale green and for surface mutants is shown in dark green. The Φ-values calculated using equilibrium for interface mutants is shown in pink and for surface mutants is shown in red. The * on top of the bar highlights the solvent exposed (Ala-Gly) mutations in the bar chart.
